# Magnetic Tunneling Between Disc-Shaped Obstacles

**DOI:** 10.1007/s00220-025-05295-5

**Published:** 2025-04-21

**Authors:** Søren Fournais, Léo Morin

**Affiliations:** Department of Mathematics, University of Copenhagen, Universitetsparken 5, 2100 Copenhagen Ø, Denmark

## Abstract

In this paper we derive formulae for the semiclassical tunneling in the presence of a constant magnetic field in 2 dimensions. The ‘wells’ in the problem are identical discs with Neumann boundary conditions, so we study the magnetic Neumann Laplacian in the complement of a set of discs. We provide a reduction method to an interaction matrix, which works for a general configuration of obstacles. When there are two discs, we deduce an asymptotic formula for the spectral gap. When the discs are placed along a regular lattice, we derive an effective operator which gives rise to the famous Harper’s equation. Main challenges in this problem compared to recent results on magnetic tunneling are the fact that one-well ground states have non-trivial angular momentum which depends on the semiclassical parameter, and the existence of eigenvalue crossings.

## Main Results

### Introduction

This article is devoted to the spectral analysis of the magnetic Laplacian

$$\begin{aligned} {\mathscr {L}}_{h}= \Big (-ih \partial _{x_1} + \frac{B x_2}{2} \Big )^2 + \Big (-ih \partial _{x_2} - \frac{B x_1}{2} \Big )^2, \end{aligned}$$

acting on domains of ${\mathbb {R}}^2$ of the form

$$\begin{aligned} \Omega _V = {\mathbb {R}}^2{\setminus }\Big ( \bigcup _{\alpha \in V} D(\alpha ,R) \Big ), \end{aligned}$$

where $R>0$ is a fixed radius, and *V* is a discrete set of points. We assume that the obstacles $D(\alpha ,R)$ are disjoint, and we denote by ${\mathscr {L}}_{h}^V$ the magnetic Neumann realization of ${\mathscr {L}}_{h}$ on $\Omega _V$.

When the magnetic field is very large—which is equivalent to the semiclassical limit $h \rightarrow 0^+$—we know that there are bound states concentrating along the boundaries of the obstacles, which decay exponentially fast in the distance to the obstacles. In fact, the low-lying spectrum of ${\mathscr {L}}_{h}^V$ is a superposition of the spectra of each single-obstacle problem^1^${\mathscr {L}}_{h}^\alpha$, up to exponentially small corrections. The aim of this article is to estimate these small terms, which are due to the interactions between the obstacles. This is an example of the tunneling effect in the presence of a magnetic field. Tunneling with magnetic field has given rise to recent investigations in various contexts. It is especially interesting because the magnetic field affects tunneling in a different way than the standard tunneling between potential wells, which is well understood and explained in [11, 15] for instance. Magnetic tunneling is only fully understood in few cases, and only in dimension two. In several models, tunneling occurs along a curve on which an effective one dimensional operator can be derived (see [3] along the boundary of a domain, [8] along a discontinuity curve of the magnetic field, [1] along a vanishing curve of the magnetic field). Other works [5, 9, 13, 14, 20] rely on radiality assumptions on the single well problem. This article is inspired by [9, 20] where a sharp tunneling formula—i.e. an estimate of the spectral gap—between two radial magnetic wells is obtained. The non radial situation is still not understood.

Frank [10] considered a similar problem of magnetic tunneling between obstacles. He deals with non-radial obstacles which are distributed along a periodic lattice, and obtains an upper bound on tunneling which is exponentially small but not sharp. One of the difficulties in this setting is the infinite number of interactions. His work was another motivation for us, and we indeed prove below sharp tunneling estimates in the case of disks, using the methods of [9, 20] which are inspired by [15]. A related problem is the tight-binding reduction studied in [23]. Some of our techniques to deal with infinitely many obstacles are inspired by [10, 23]. Note that, as in [23], our analysis allows for arbitrary configurations of obstacles, which do not have to satisfy any kind of periodicity.

The case of periodic obstacles is also similar to the setting of [16], where the tunneling is induced by a periodic potential in the presence of a homogeneous magnetic field. However in that work, the magnetic field has to be small enough, so that the tunneling effect is dominated by the potential. In [16] it is also underlined how this continuous periodic model is effectively described by the Harper equation (a special case of the Almost-Mathieu operator). We recall this link in Sect. 1.4 below.

Let us also mention the recent work [12], where tunneling between two radial electric wells with Aharonov–Bohm potential is studied. In this setting, the one-well operator has the same structure as ours. The tunneling formula is similar to our Theorems 3 and 4. However, the authors do not consider more than two wells.

Finally, a first tunneling formula between non-radial magnetic wells was recently obtained in [6], where a new approach to magnetic tunneling is also developed. Even though these techniques seem robust, it is not clear how to adapt them to our setting. In this paper, we also illustrate other aspects of tunneling that are absent in [6]: Firstly, we allow the single-obstacle problem to have multiplicities, and secondly we can deal with an arbitrary number of obstacles. These aspects require an extra careful analysis.

Estimating the interaction between the obstacles $\{ D(\alpha ,R)\}_{\alpha \in V}$ requires a good understanding of the single-obstacle operator ${\mathscr {L}}_{h}^\alpha$. First of all, these operators are unitarily equivalent to each other. Indeed, if $\tau _\alpha ^B$ denotes the magnetic translation acting on $\Psi \in L^2({\mathbb {R}}^2)$ as^2^

$$\begin{aligned} \tau _\alpha ^B \Psi (x) = e^{\frac{iB}{2h} \alpha \wedge x} \Psi (x-\alpha ), \end{aligned}$$

then we have

1.1 $$\begin{aligned} (\tau _\alpha ^B)^* {\mathscr {L}}_{h}^\alpha \tau _\alpha ^B = {\mathscr {L}}_{h}^0. \end{aligned}$$

We also mention the main properties of $\tau _\alpha ^B$,

$$\begin{aligned} \tau _\alpha ^B \tau _\beta ^B = e^{\frac{iB}{2h} \alpha \wedge \beta } \tau _{\alpha +\beta }^B = e^{\frac{iB}{h} \alpha \wedge \beta } \tau _{\beta }^B \tau _\alpha ^B, \quad \mathrm{{and}} \quad (\tau _\alpha ^B)^* = \tau _{-\alpha }^B. \end{aligned}$$

From (1.1) we deduce that ${\mathscr {L}}_{h}^\alpha$ has the same spectrum as ${\mathscr {L}}_{h}^0$ and that the corresponding eigenfunctions are related by magnetic translations. The spectrum of ${\mathscr {L}}_{h}^0$ has non-trivial dependence on *B*—or equivalently on *h*. The first eigenvalues are described in [7]. They depend on the following oscillating function of *h*,

1.2 $$\begin{aligned} e(h) = \inf _{m \in {\mathbb {Z}}} | \xi _h - m |, \qquad \xi _h = \frac{BR^2}{2 h} + \sqrt{\frac{\Theta _0 BR^2}{ h}} + {\mathcal {C}}_2, \end{aligned}$$

where $\Theta _0 \in (0,1)$ and ${\mathcal {C}}_2 \in {\mathbb {R}}$ are universal constants. To emphasize this dependence, we introduce the following notion.

#### Definition 1

Let $e_0 \in (-\frac{1}{2}, \frac{1}{2}]$. We say that $h \rightarrow 0$ along an $e_0$-sequence if *h* follows a sequence $(h_n)_{n \in {\mathbb {N}}}$ with $\lim _{n \rightarrow 0} h_n = 0$, such that

$$\begin{aligned} \xi _{h_n} - n \underset{n \rightarrow \infty }{\longrightarrow }\ e_0. \end{aligned}$$

In particular, if $h \rightarrow 0$ along an $e_0$-sequence then $e(h) \rightarrow |e_0|$. With this notation we have the following description of the first eigenvalues of ${\mathscr {L}}_{h}^0$.

#### Theorem 2

([7] Spectrum of the single-obstacle operator). There exist universal constants $\Theta _0 \in (0,1)$, ${\mathcal {C}}_1 >0$ and ${\mathcal {C}}_0$, ${\mathcal {C}}_2 \in {\mathbb {R}}$ such that the following holds. For all $B,R>0$ and $e_0 \in (-\frac{1}{2}, \frac{1}{2}]$ we have:

1. The first eigenvalue $\mu _1$ of ${\mathscr {L}}_{h}^0$ satisfies
  $$\begin{aligned} \mu _1 = \Theta _0 B h + \frac{ {\mathcal {C}}_1 \sqrt{B}}{R} h^{\frac{3}{2}} + \frac{3 {\mathcal {C}}_1 \sqrt{\Theta _0}}{R^2} h^2 \big ( e_0^2 + {\mathcal {C}}_0 \big ) + o(h^2), \end{aligned}$$
  when $h \rightarrow 0$ along an $e_0$-sequence.
2. The second eigenvalue $\mu _2$ of ${\mathscr {L}}_{h}^0$ satisfies
  $$\begin{aligned} \mu _2 = \Theta _0 B h + \frac{ {\mathcal {C}}_1 \sqrt{B}}{R} h^{\frac{3}{2}} + \frac{3 {\mathcal {C}}_1 \sqrt{\Theta _0}}{R^2} h^2 \big ( (1-|e_0|)^2 + {\mathcal {C}}_0 \big ) + o(h^2), \end{aligned}$$
  when $h \rightarrow 0$ along an $e_0$-sequence.
3. The third eigenvalue $\mu _3$ of ${\mathscr {L}}_{h}^0$ satisfies
  $$\begin{aligned} \mu _3 = \Theta _0 B h + \frac{ {\mathcal {C}}_1 \sqrt{B}}{R} h^{\frac{3}{2}} + \frac{3 {\mathcal {C}}_1 \sqrt{\Theta _0}}{R^2} h^2 \big ( (1+|e_0|)^2 + {\mathcal {C}}_0 \big ) + o(h^2), \end{aligned}$$
  when $h \rightarrow 0$ along an $e_0$-sequence.

Note that $|e_0|$, $1-|e_0|$ and $1+|e_0|$ are respectively the distance of $\xi _h$ to the closest, second closest, and third closest integer.

### Tunneling between two obstacles

Starting from Theorem 2, we can study the interaction between two discs. We denote by $L>2R$ the distance between two centers $\alpha _\ell = \big ( - \frac{L}{2},0 \big )$ and $\alpha _r = \big ( \frac{L}{2},0 \big )$, and we consider ${\mathscr {L}}_{h}^V$ with $V= \lbrace \alpha _\ell , \alpha _r \rbrace$. If $|e_0| \ne \frac{1}{2}$, then the first two eigenvalues of ${\mathscr {L}}_{h}^0$ are separated by a spectral gap of order $h^2$ and thus the situation is similar to [9, 20]: ${\mathscr {L}}_{h}^V$ has two eigenvalues of order $\mu _1$ which are exponentially close to each other.

#### Theorem 3

(Two obstacles with $|e_0|\ne \frac{1}{2}$). Let $B,R,L>0$ and $e_0 \in (-\frac{1}{2}, \frac{1}{2})$. Assume $V = \lbrace \alpha _\ell , \alpha _r \rbrace$ and $L>6R$. Define

1.3 $$\begin{aligned} S_h(L) = \frac{BL}{4}\sqrt{L^2 - 4R^2} - \big ( BR^2 + 2\sqrt{h\Theta _0 BR^2} \big ) \ln \Big ( \frac{L + \sqrt{L^2 - 4R^2}}{2R}\Big ) \end{aligned}$$

and

$$\begin{aligned} c_0 = \frac{3 {\mathcal {C}}_1\sqrt{\Theta _0}}{R^2}. \end{aligned}$$

There exist $C=C(B,R,L) >0$ and $K = K(B,R,L)>0$ such that the following holds. If *h* is small enough along an $e_0$-sequence, the spectrum of ${\mathscr {L}}_{h}^V$ in $(- \infty , \mu _1 + \frac{c_0(1-2|e_0|)}{2} h^2 )$ consists of exactly two eigenvalues $\lambda _1 < \lambda _2$ such that

$$\begin{aligned} \lambda _2 - \lambda _1= 2C K^{-2e_0} h e^{-\frac{S_h(L)}{h}}(1+ o(1)), \end{aligned}$$

as $h\rightarrow 0$ along an $e_0$-sequence.

*Remark*. The factor 6 in the condition $L > 6R$ is a technical artifact of our proof. We expect tunneling to occur for any $L>2R$. However, the magnetic tunneling has an oscillating component on top of an exponential decay, and both contribute to the final $S_h$. In the proof, one needs to control that an error term where the oscillations are eliminated remains smaller than the main term. This requires the obstacles to be sufficiently far from each other. The estimate in question is considered in Appendix C.

Similar conditions occur in the following Theorems 4 and 5 (with a constant 25 instead of 6). The reason remains the same.

When $|e_0|=\frac{1}{2}$, the situation is more complicated because the first eigenvalue $\mu _1$ may become double. In fact, it is known that $\mu _1$ is double for some arbitrarily small values of *h*. However, in this case there is a gap of order $h^2$ between the first two eigenvalues $\{\mu _1,\mu _2\}$ and the third eigenvalue. Using this gap, we can prove that there are four eigenvalues which are exponentially close to each other.

#### Theorem 4

(Two obstacles with $e_0 = \frac{1}{2}$). Let $B,R,L>0$ with $L> 6R$ and assume $V = \lbrace \alpha _\ell , \alpha _r \rbrace$. Let $h>0$ be small enough and such that $\mu _1(h) = \mu _2(h)$. Then the spectrum of ${\mathscr {L}}_{h}^V$ in $(- \infty , \mu _1 + c_0 h^2 )$ consists of four eigenvalues $\lambda _1 \le \lambda _2 \le \lambda _3 \le \lambda _4$ such that

$$\begin{aligned} \lambda _2 - \lambda _1&= C ( K^{\frac{1}{2}} + K^{- \frac{3}{2}}) h e^{- \frac{S_h(L)}{h}}( 1+o(1)),\\ \lambda _3 - \lambda _2&= o \Big ( h e^{- \frac{S_h(L)}{h}} \Big ),\\ \lambda _4 - \lambda _3&= C ( K^{\frac{1}{2}} + K^{- \frac{3}{2}}) h e^{- \frac{S_h(L)}{h}} ( 1+o(1)), \end{aligned}$$

with the same constants $C, K>0$ and $c_0>0$ as in Theorem 3.

*Remark*. Note that the assumption $\mu _1=\mu _2$ implies that $|e_0|=\frac{1}{2}$ by Theorem 2. In this case, there is no reason for the eigenvalues of ${\mathscr {L}}_{h}^V$ not to be double, since this is the case for the single obstacle. It is remarkable that for the double well operator, there is still a gap between $\lambda _2$ and $\lambda _1$. It is a natural question whether $\lambda _2 = \lambda _3$ or not. It could be that some hidden symmetry of ${\mathscr {L}}_{h}^0$ is somehow inherited by ${\mathscr {L}}_{h}^V$.

### Periodic obstacles

Another interesting situation, we can deal with, is the case of obstacles on a square lattice, as in [10]. When $V= L {\mathbb {Z}}^2$, ${\mathscr {L}}_{h}^V$ has infinitely many eigenvalues of order $\mu _1$. In Theorem 5 below, we prove that this part of the spectrum is given by an effective operator ${\textbf{X}}$ on $\ell ^2(L {\mathbb {Z}}^2)$, with coefficients

$$\begin{aligned} {\textbf{X}}_{\alpha \beta } = {\left\{ \begin{array}{ll} e^{\frac{iB}{2h}\alpha \wedge \beta }, & \quad {\text {if}} \; |\alpha - \beta |=L,\\ 0, & \quad {\text {otherwise}}. \end{array}\right. } \end{aligned}$$

In the case $e_0 = \frac{1}{2}$, the effective operator ${\textbf{Y}}$ acts on $\ell ^2(L{\mathbb {Z}}^2) \otimes {\mathbb {C}}^2$ with coefficients

$$\begin{aligned} {\textbf{Y}}_{\alpha \beta }^{\sigma \sigma '}= {\left\{ \begin{array}{ll} - \sigma ' e^{\frac{iB}{2h}\alpha \wedge \beta } e^{- i \frac{\sigma - \sigma '}{2} \arg (\beta -\alpha )} K^{\frac{\sigma + \sigma '}{2}},& \quad {\textrm{if}} \, |\alpha - \beta |=L, \\ 0, & \quad {\textrm{otherwise,}} \end{array}\right. } \end{aligned}$$

for $\alpha$, $\beta \in L{\mathbb {Z}}^2$ and $\sigma$, $\sigma ' \in \lbrace \pm \rbrace$. More precisely, we obtain the following result.

#### Theorem 5

(Obstacles on a square lattice). Let *B*, *R*, $L>0$ and $e_0 \in (- \frac{1}{2}, \frac{1}{2}]$. Assume $V = L {\mathbb {Z}}^2$, and $L> 25 R$. For $t>0$ define the spectral subspace

$$\begin{aligned} {\mathscr {E}}(t) = {\textbf{1}}_{(-\infty , \mu _1 + th^2]} ( {\mathscr {L}}_{h}^V ). \end{aligned}$$

There exist $C=C(B,R,L) >0$ and $K = K(B,R,L)>0$ such that the following holds. Assume that $h \rightarrow 0$ along an $e_0$-sequence.

1. If $e_0 \ne \frac{1}{2}$, then for *h* small enough, the restriction of ${\mathscr {L}}_{h}^V$ to ${\mathscr {E}}\big ( c_0 \frac{1-2e_0}{2} \big )$ is unitarily equivalent to a bounded operator ${\textbf{M}}$ on $\ell ^2(L{\mathbb {Z}}^2)$ such that
  $$\begin{aligned} {\textbf{M}}= \mu _1 {\textbf{I}} + \lambda {\textbf{X}}+ o(h e^{-\frac{S_h(L)}{h}}), \end{aligned}$$
  where $$|\lambda | = 2C K^{-2e_0} h e^{- \frac{S_h(L)}{h}}$$.
2. If $e_0= \frac{1}{2}$, then we assume furthermore that $\mu _1(h) = \mu _2(h)$ along the $e_0$-sequence. Then for *h* small enough, the restriction of ${\mathscr {L}}_{h}^V$ to ${\mathscr {E}}(c_0)$ is unitarily equivalent to a bounded operator ${\textbf{M}}$ on $\ell ^2(L {\mathbb {Z}}^2) \otimes {\textbf{C}}^2$ such that
  $$\begin{aligned} {\textbf{M}}= \mu _1 {\textbf{I}} + \lambda {\textbf{Y}}+ o(h e^{-\frac{S_h(L)}{h}}), \end{aligned}$$
  where $$\lambda = (-1)^{\lfloor \xi _h \rfloor } 2 C K^{- \frac{1}{2}} h e^{- \frac{S_h(L)}{h}}$$.

In both cases the operator ${\textbf{M}}$ is obtained by constructing an eigenbasis for ${\mathscr {L}}_{h}^V$ starting from the ground states of each single-obstacle operator ${\mathscr {L}}_{h}^\alpha$ (for $\alpha \in L {\mathbb {Z}}^2$).

### Link with the Almost-Mathieu operator (or Harper equation)

The effective operator ${\textbf{X}}$ from Theorem 5 is in fact unitarily equivalent to a direct integral of Almost-Mathieu operators. Indeed, we can first conjugate ${\textbf{X}}$ by the unitary diagonal matrix $\mathrm{{diag}} \big ( e^{i \frac{B}{2\,h} \alpha _1 \alpha _2} \big )_{\alpha \in V}$. Thus ${\textbf{X}}$ is unitarily equivalent to ${\textbf{X}}'$ with

$$\begin{aligned} {\textbf{X}}'_{\alpha \beta } = e^{i \frac{B}{2h}(\beta _1 + \alpha _1)(\beta _2 - \alpha _2)}, \qquad |\alpha -\beta |=L \end{aligned}$$

This is a convolution in the variable $\alpha _2$, and it can be diagonalized using the following Fourier transform,

$$\begin{aligned} ({\mathscr {F}} f )(\alpha _1,\alpha _2) = \frac{1}{\sqrt{2\pi }}\int _{0}^{2\pi } e^{-i \theta \alpha _2 /L } f_{\alpha _1}(\theta ) \textrm{d}\theta , \quad f \in \ell ^2(L {\mathbb {Z}}) \otimes L^2(0,2\pi ), \end{aligned}$$

with inverse

$$\begin{aligned} ({\mathscr {F}}^{-1} g)(\alpha _1,\theta ) = \frac{1}{\sqrt{2\pi }} \sum _{\alpha _2 \in L {\mathbb {Z}}} e^{i \theta \alpha _2 /L} g_{(\alpha _1,\alpha _2)}, \quad g \in \ell ^2(L {\mathbb {Z}}^2). \end{aligned}$$

Then we have for all $f \in \ell ^2(L{\mathbb {Z}}) \otimes L^2(0,2\pi )$,

$$\begin{aligned} ({\mathscr {F}}^{-1} {\textbf{X}}' {\mathscr {F}} f)(\alpha _1, \theta ) = f(\alpha _1 - L, \theta ) + f(\alpha _1+L,\theta ) + 2 \cos \big ( \theta - \frac{BL}{h} \alpha _1 \big ) f(\alpha _1,\theta ). \end{aligned}$$

Therefore,

$$\begin{aligned} {\mathscr {F}}^{-1} {\textbf{X}}' {\mathscr {F}} = \int _{(0,2\pi )}^\oplus {\mathscr {H}}_{\theta , \frac{BL^2}{2 \pi h}} \textrm{d}\theta , \end{aligned}$$

where ${\mathscr {H}}_{\theta ,\phi }$ is the Almost-Mathieu (or Harper) operator on $\ell ^2({\mathbb {Z}})$,

$$\begin{aligned} ({\mathscr {H}}_{\theta ,\phi } u)(n) = u(n-1) + u(n+1) +2 \cos (\theta + 2 \pi n \phi ) u(n). \end{aligned}$$

This operator has been object of many works (see for instance [2, 17, 18, 21], and the recent surveys [19, 22]). It is well-known to have very singular dependence on $\phi$. In particular, the following properties can be transfered to ${\textbf{X}}$.

- If $\phi$ is rational, the spectrum of ${\mathscr {H}}_{\theta ,\phi }$ is purely absolutely continuous and independent of $\theta$.
- If $\phi$ is irrational, the spectrum of ${\mathscr {H}}_{\theta ,\phi }$ is purely singular continuous [18].
- If $\phi$ is irrational, the spectrum of ${\mathscr {H}}_{\theta ,\phi }$ is a Cantor set with Lebesgue measure 0 [2].

The picture of the spectrum of ${\mathscr {H}}_{\theta ,\phi }$ as function of $\phi$ leads to the famous Hofstadter’s butterfly. Theorem 5 therefore states that, around $\mu _1$, the spectrum of ${\mathscr {L}}_{h}^V$ is given to main order by a Hofstadter’s butterfly (as function of the flux $\phi =\frac{BL^2}{2\pi h}$).

*Remark*. Near the crossing points $\mu _1 = \mu _2$, Theorem 5 gives the effective operator ${\textbf{Y}}$, which is more complicated than an Almost-Mathieu operator, since it involves an effective spin. Using the same transformations as for ${\textbf{X}}$, we find that ${\textbf{Y}}$ is conjugated to the operator

$$\begin{aligned} \int _{(0,2\pi )}^\oplus {\mathscr {H}}'_{\theta , \frac{BL^2}{2\pi h}} \textrm{d}\theta , \end{aligned}$$

where ${\mathscr {H}}_{\theta ,\phi }': \ell ^2({\mathbb {Z}}) \otimes {\mathbb {C}}^2 \rightarrow \ell ^2({\mathbb {Z}}) \otimes {\mathbb {C}}^2$ is defined by

$$\begin{aligned} ({\mathscr {H}}_{\theta ,\phi }' u)(n) = {\mathcal {T}} u(n+1) + {\mathcal {T}}^* u(n-1) + 2 {\mathcal {A}}_n u(n), \end{aligned}$$

with

$$\begin{aligned} {\mathcal {T}} = \begin{pmatrix} -K & \quad 1 \\ -1 & \quad K^{-1} \end{pmatrix}, \quad {\mathcal {A}}_n = \begin{pmatrix} - K \cos (2 \pi n \phi - \theta ) & \quad \sin (2\pi n \phi - \theta ) \\ \sin (2\pi n \phi - \theta ) & \quad K^{-1} \cos (2\pi n \phi - \theta ) \end{pmatrix}. \end{aligned}$$

This operator is maybe linked to the unitary Almost-Mathieu operators studied in [4] for instance.

### Organization of the paper

The analysis necessary for the proofs of all main theorems: Theorems 3, 4, and 5, is to a large extent overlapping: The first two theorems concern the case of 2 obstacles and in the last the set of obstacles is an infinite lattice. Therefore, in Sects. 2–4 we carry through this analysis in a unified way for a general set of obstacles. The first Sect. 2 contains the relevant information about the one-obstacle problem, which is an extremely important input to the rest of the paper. A main observation is that using the rotational symmetry of the one-obstacle problem, one can understand ground states in terms of a special function—the Kummer function—in the radial variable. A similar insight was used in [5] and spurred much progress on special cases of tunneling with magnetic fields (see in particular [9, 13, 14, 20]), however a main novelty in the present work is the presence of non-zero, *h*-dependent, angular momentum in the ground state and also the accompanying crossing of eigenvalues. Sections 3 and 4 both contain the reduction to an interaction matrix, in the cases without eigenvalue crossing ($e_0 \ne \frac{1}{2}$) and near a crossing ($e_0 = \frac{1}{2}$) respectively. All of this analysis is for a general set *V* of obstacles and therefore include both the case of only two obstacles and the case of an infinite lattice of them. Note also, that the periodicity of the obstacles in the case of infinitely many is not used in this part of the paper.

The Sect. 5 now gives the proofs of Theorems 3 and 4, i.e. the tunneling formulas for two obstacles in the cases away from crossings or exactly at the crossings respectively. Section 6 contains the tunneling analysis for an infinite set of obstacles placed on a regular lattice.

The most computational parts of the analysis are delegated to the appendices, in order to lighten the rest of the presentation. However, these parts are also fundamental for the final estimates. In Appendix A, we gather the main properties of the phases involved in several integrals, needed to use the Laplace method. In Appendix B, we calculate the interaction coefficients arising in the effective operators. Finally, we bound some error terms in Appendix C.

## The Single Obstacle Operator ${\mathscr {L}}_{h}^0$

### Fourier decomposition

We gave a description of the first eigenvalues of ${\mathscr {L}}_{h}^0$ in Theorem 2. Let us recall some further properties of this operator. Since we consider magnetic-Neumann boundary conditions, the domain of ${\mathscr {L}}_{h}^0$ is

$$\begin{aligned} \mathrm{{Dom}} \big ( {\mathscr {L}}_{h}^0 \big ) = \lbrace \Psi \in H^1(\Omega ^0): \quad {\mathscr {L}}_{h}\Psi \in L^2(\Omega ^0), \quad \partial _r \Psi = 0 \quad \mathrm{{on }} \; \partial D(0,R) \rbrace , \end{aligned}$$

where we recall that $\Omega ^0 = {\mathbb {R}}^2 {\setminus } D(0,R)$. Here $\partial _r$ denotes the radial derivative. In radial coordinates $(r,\theta )$ the differential operator ${\mathscr {L}}_{h}$ is given by^3^

2.1 $$\begin{aligned} {\mathscr {L}}_{h}= - \frac{h^2}{r} \partial _r r \partial _r + \frac{1}{r^2}\Big (-ih \partial _\theta - \frac{B r^2}{2} \Big )^2. \end{aligned}$$

In particular, ${\mathscr {L}}_{h}^0$ commutes with $\partial _\theta$ and we can decompose according to Fourier modes. Therefore, it is unitarily equivalent to the direct sum of the family of operators

2.2 $$\begin{aligned} {\mathscr {L}}_{h,m} =- \frac{h^2}{r} \partial _r r \partial _r + \frac{1}{r^2}\Big (h m - \frac{B r^2}{2} \Big )^2, \qquad m \in {\mathbb {Z}}. \end{aligned}$$

For all radial functions *f* we have ${\mathscr {L}}_{h,m} f = e^{im \theta } {\mathscr {L}}_{h}^0 e^{-im\theta } f$. Thus, the spectrum of ${\mathscr {L}}_{h}^0$ is the union of the spectra of each ${\mathscr {L}}_{h,m}$. A more precise result follows from the discussion in [7] on the first eigenvalues.

#### Lemma 6

Let $$e_0 \in (-\frac{1}{2}, \frac{1}{2}]$$.

1. If *h* is small enough along an $e_0$-sequence with $e_0 \ne \frac{1}{2}$, there is a unique integer $m_-$ closest to $\xi _h$, and it satisfies
  $$\begin{aligned} m_- = \xi _h - e_0 + o(1). \end{aligned}$$
  With this *m*, the ground state energy of ${\mathscr {L}}_{h}^0$ is
  $$\begin{aligned} \mu _1 = \lambda _1( {\mathscr {L}}_{h,m_-}). \end{aligned}$$
2. If *h* is small enough along a $\frac{1}{2}$-sequence, the two closest integers $m_-$ and $m_+$ to $\xi _h$ satisfy
  $$\begin{aligned} {\left\{ \begin{array}{ll} m_- = \xi _h - \frac{1}{2} + o(1) \\ m_+ = \xi _h + \frac{1}{2} + o(1). \end{array}\right. } \end{aligned}$$
  Which one is the closest depends on *h*. The two smallest eigenvalues of ${\mathscr {L}}_{h}^0$ are given by
  $$\begin{aligned} \lbrace \mu _1, \mu _2 \rbrace = \lbrace \mu _-, \mu _+ \rbrace , \end{aligned}$$
  where $$\mu _{\pm } = \lambda _1( {\mathscr {L}}_{h,m_\pm })$$.

In any case, we will always denote by $\Phi ^{-}$ and $\Phi ^+$ the eigenfunctions of ${\mathscr {L}}_{h}^0$ with respective momenta $m_-$ and $m_+ = m_- +1$. In other words,

2.3 $$\begin{aligned} e^{-im_{\pm } \theta } \Phi ^\pm \quad {\text {is the ground state of}} \quad {\mathscr {L}}_{h,m_\pm }. \end{aligned}$$

We denote by $\mu _\pm$ the associated eigenvalue.

### Decay of the eigenfunctions

A fundamental aspect of our analysis is that we know precisely how fast the eigenfunctions $\Phi ^\pm$ decay away from $r=R$. The decay rate is given by the function

2.4 $$\begin{aligned} d(r) = \frac{B}{4}(r^2 - R^2) - \frac{BR^2}{2} \ln ( r/R), \end{aligned}$$

which we call *Agmon distance* by analogy with the case of potential wells. Indeed, one has

2.5 $$\begin{aligned} |\Phi ^\pm (r) | \le C_\eta e^{- \frac{(1-\eta ) d(r)}{h}}, \end{aligned}$$

for all $\eta >0$. We use these bounds all through the paper. They are consequences of the following lemma, recalling that $m_\pm \sim \frac{BR^2}{2\,h}$ as $h \rightarrow 0$.

#### Lemma 7

The eigenfunctions $\Phi ^\pm$ of ${\mathscr {L}}_{h}^0$, defined in (2.3), are of the form

2.6 $$\begin{aligned} \Phi ^\pm (r,\theta ) = \Big ( \frac{BR^2}{h} \Big )^{\frac{\Theta _0}{4}} \Big ( \frac{re^{i\theta }}{R} \Big )^{m_{\pm }} e^{- \frac{B}{4h}(r^2 - R^2)} u_h(r), \end{aligned}$$

where $u_h$ is a real-analytic function on ${{\textrm{Re}}}(r^2) >0$, which is locally uniformly bounded with respect to *h*. Moreover,

- For all *r* such that ${{\textrm{Re}}}(r^2) >R^2$, $u_h(r)$ is asymptotically given by
  2.7 $$\begin{aligned} u_h(r) = \frac{K_0 \Gamma (\delta _0)}{R} \Big ( \frac{2R^2}{r^2-R^2} \Big )^{\delta _0} \Big ( 1 + o \Big ( \frac{h^{\frac{1}{2}}}{r^2-R^2} \Big ) \Big ), \end{aligned}$$
  as $h \rightarrow 0$, where $\delta _0 = \frac{1 - \Theta _0}{2}$ and $K_0>0$ is a universal constant.
- For all $t>0$ there is a $v(t) >0$ such that
  2.8 $$\begin{aligned} u_h \Big (R+ t \sqrt{\frac{h}{B}} \Big ) = \frac{K_0 v(t)}{R} \Big ( \frac{h}{BR^2} \Big )^{\frac{\Theta _0 - 1}{4}} \big ( 1 +o(1) \big ) \end{aligned}$$
  as $h \rightarrow 0$. In fact, *v* is a solution to
  $$\begin{aligned} -v''(t) + 2(t- \sqrt{\Theta _0}) v'(t) + v(t) = \Theta _0 v(t). \end{aligned}$$
- For all $$t>0$$,
  2.9 $$\begin{aligned} \partial _r u_h\Big (R+t \sqrt{\frac{h}{B}} \Big ) = \frac{K_0 v'(t)}{ R^2} \Big ( \frac{h}{BR^2} \Big )^{\frac{\Theta _0 - 3}{4}} \big ( 1+ o(1) \big ) \end{aligned}$$
  as $$h \rightarrow 0$$.

Note that we do not use any WKB expansion in the proof of this Lemma: In fact, the eigenfunctions can be written using special functions.

#### Proof

We have an explicit formula for $\Phi ^\pm$ in terms of the Kummer function,

$$\begin{aligned} \Phi ^{\pm }(r,\theta ) = C_{\pm } e^{im_{\pm } \theta } r^{m_{\pm }} \int _0^\infty e^{- \frac{B r^2}{4h}(1+2s)} (1+s)^{m_{\pm } - \delta _\pm } s^{\delta _\pm -1} ds, \end{aligned}$$

where $\delta _\pm = \frac{1}{2} - \frac{\mu _{\pm }}{2 B h}$, and $C_{\pm } \ne 0$ is an *h*-dependent normalization constant. This formula can be derived as in [9], but one can also check directly that it is indeed a solution to the eigenvalue equation. In particular, $\Phi ^\pm$ has the form (2.6), with

2.10 $$\begin{aligned} u_h(r) = C_\pm R^{m_\pm } \Big ( \frac{h}{BR^2}\Big )^{\frac{\Theta _0}{4}} e^{- \frac{BR^2}{4h}} \int _0^\infty e^{- \frac{Bs r^2}{2h} + m_\pm \ln ( 1+s)} \frac{s^{\delta _\pm - 1}}{(1+s)^{\delta _\pm }} \textrm{d}s. \end{aligned}$$

The remaining of the proof consists in estimating $u_h$ and $C_\pm$ using the Laplace method. Note that the phase involves the two different frequencies *h* and $\sqrt{h}$ via $m_{\pm }$.

1. To estimate $u_h(r)$, we start by the substitution $s = \frac{h}{BR^2} s'$ suggested by the Laplace method, which gives

$$\begin{aligned} u_h(r) = C_\pm R^{m_\pm } \Big ( \frac{h}{BR^2}\Big )^{\frac{\Theta _0}{4} + \delta _\pm } e^{- \frac{BR^2}{4h}} \int _0^\infty e^{- \frac{s r^2}{2R^2} + m_\pm \ln \big ( 1+ \frac{h}{BR^2} s \big )} s^{\delta _\pm - 1} \big (1+ {\mathcal {O}} ( h s) \big ) \textrm{d}s. \end{aligned}$$

The involved phase satisfies

$$\begin{aligned} \frac{sr^2}{2R^2} - m_{\pm }(h) \ln \big ( 1+ \frac{h}{BR^2} s \big ) = \frac{ (r^2 - R^2) s}{2R^2} + {\mathscr {O}}(\sqrt{h}s). \end{aligned}$$

Therefore, the Laplace method gives

2.11 $$\begin{aligned} u_h(r) = C_\pm R^{m_\pm } \Big ( \frac{h}{BR^2}\Big )^{ \frac{1}{2} - \frac{\Theta _0}{4}} e^{- \frac{BR^2}{4h}} \Big ( \frac{2 R^2}{ r^2-R^2} \Big )^{\delta _0} \Gamma ( \delta _0) \Big ( 1 + o \Big ( \frac{h^{\frac{1}{2}}}{r^2-R^2} \Big ) \Big ),\nonumber \\ \end{aligned}$$

where we also replaced $\delta _\pm$ by its asymptotic value $\delta _0 = \frac{1 - \Theta _0}{2}$.

2. Near $r=R$ we need finer estimates, and we consider for any $t>0$,

$$\begin{aligned} u_h \Big (R + t \sqrt{\frac{h}{B} } \Big ) =C_\pm R^{m_\pm } \Big ( \frac{h}{BR^2}\Big )^{\frac{\Theta _0}{4}} e^{- \frac{BR^2}{4h}} \int _0^\infty e^{- \frac{Bs}{2 h} \big (R+t\sqrt{\frac{h}{B}} \big )^2 + m_{\pm } \ln (1+s)} \frac{s^{\delta _{\pm }-1}}{(1+s)^{\delta _{\pm }}} \textrm{d}s. \end{aligned}$$

This time, the Laplace method suggests the substitution $s = \sqrt{\frac{h}{BR^2}} s'$, and we obtain

$$\begin{aligned} u_h \Big (R + t \sqrt{\frac{h}{B} } \Big ) = C_\pm R^{m_\pm } \Big ( \frac{h}{BR^2}\Big )^{\frac{\Theta _0}{4} + \frac{\delta _\pm }{2}} e^{- \frac{BR^2}{4h}} \int _0^\infty e^{- f(s,t,h)} s^{\delta _\pm - 1} \big (1+ {\mathcal {O}} ( \sqrt{h} s) \big ) \textrm{d}s, \end{aligned}$$

with phase

$$\begin{aligned} f(s,t,h)&= \sqrt{\frac{BR^2}{h}} \frac{s}{2} \Big (1+ t \sqrt{\frac{h}{BR^2}} \Big )^2 - m_{\pm } \ln \Big (1+ s \sqrt{\frac{h}{BR^2}} \Big ) \\&= \sqrt{\frac{BR^2}{h}} \frac{s}{2} + ts + \frac{t^2 s}{2} \sqrt{\frac{h}{BR^2}} \\  &\qquad - \Big ( \frac{BR^2}{2h} + \sqrt{\frac{\Theta _0 BR^2}{h}} + {\mathscr {O}}(1) \Big ) \Big (s \sqrt{ \frac{h}{BR^2}} - \frac{h s^2}{2BR^2} + {\mathscr {O}}(h^{3/2}s^3) \Big ) \\&= ts + \frac{s^2}{4} - \sqrt{\Theta _0} s + {\mathscr {O}}(\sqrt{h}s(1+s^2)). \end{aligned}$$

Thus, the Laplace method gives

2.12 $$\begin{aligned} u_h \Big (R + t \sqrt{\frac{h}{B} } \Big ) = C_\pm R^{m_\pm } \Big ( \frac{h}{BR^2}\Big )^{\frac{1}{4}} e^{- \frac{BR^2}{4h}} \int _0^\infty e^{- \big ( \frac{s^2}{4} + ts - \sqrt{\Theta _0} s \big )} s^{\delta _0 - 1} \textrm{d}s \big (1+ o(1) \big ).\nonumber \\ \end{aligned}$$

3. We now estimate $C_\pm$, using that $\Vert \Phi ^\pm \Vert =1$. In polar coordinates we deduce

$$\begin{aligned} 1 =\Big ( \frac{BR^2}{h} \Big )^{\frac{\Theta _0}{2}} \int _R^\infty \Big ( \frac{r}{R} \Big )^{2 m_{\pm }} e^{- \frac{B}{2h}(r^2 - R^2)} |u_h(r)|^2 2 \pi r \textrm{d}r. \end{aligned}$$

We use the substitution $r = R + t \sqrt{\frac{h}{B}}$, and the estimate (2.12) to obtain

$$\begin{aligned} 1 = C_\pm ^2 R^{2m_\pm +1 }\Big ( \frac{h}{BR^2}\Big )^{1-\frac{\Theta _0}{2}} \int _{0}^\infty e^{- \frac{B}{2h}\big ( R + t\sqrt{\frac{h}{B}} \big )^2 + 2 m_\pm \ln \big ( 1 + t\sqrt{\frac{h}{BR^2}} \big ) } v(t)^2 2\pi R \big (1+o(1) \big ) \textrm{d}t, \end{aligned}$$

where

$$\begin{aligned} v(t) = \int _0^\infty e^{- \big ( \frac{s^2}{4} + ts - \sqrt{\Theta _0} s \big )} s^{\delta _0 - 1} \textrm{d}s. \end{aligned}$$

The involved phase satisfies

$$\begin{aligned} \frac{B}{2h}\Big (R + t\sqrt{\frac{h}{B}} \Big )^2 -2m_{\pm } \ln \Big (1+ t\sqrt{\frac{h}{BR^2}}\Big ) = \frac{BR^2}{2h} + t^2 - 2 t \sqrt{\Theta _0} + {\mathscr {O}}(\sqrt{h}(1+t^3)). \end{aligned}$$

Thus, the Laplace method gives

$$\begin{aligned} 1 = C_\pm ^2 R^{2 m_\pm +2 } \Big ( \frac{h}{BR^2}\Big )^{1-\frac{\Theta _0}{2}} e^{- \frac{BR^2}{2h}} 2 \pi \int _0^\infty e^{- \big ( t^2 - 2t \sqrt{\Theta _0} \big )} v(t)^2 \textrm{d}t \big ( 1+ o(1) \big ). \end{aligned}$$

Therefore we find

2.13 $$\begin{aligned} C_\pm = K_0 R^{-m_\pm -1} \Big ( \frac{h}{BR^2}\Big )^{\frac{\Theta _0}{4}-\frac{1}{2}} e^{\frac{BR^2}{4h}} \big ( 1+o(1) \big ), \end{aligned}$$

where $K_0^{-2}= 2\pi \int _0^\infty e^{- \big ( t^2 - 2t \sqrt{\Theta _0} \big )} v(t)^2 \textrm{d}t$. Now, we can insert (2.13) in (2.11) and (2.12) to find (2.7) and (2.8).

4. Coming back to (2.10) and differentiating we have

$$\begin{aligned} \partial _r u_h(r) = - C_\pm R^{m_\pm } \frac{ B r }{h} \Big ( \frac{h}{BR^2}\Big )^{\frac{\Theta _0}{4}} e^{- \frac{BR^2}{4h}} \int _0^\infty e^{- \frac{Bs r^2}{2h} + m_\pm \ln ( 1+s)} \frac{s^{\delta _\pm }}{(1+s)^{\delta _\pm }} \textrm{d}s. \end{aligned}$$

Therefore, similarly to (2.12) we find

$$\begin{aligned} \partial _r u_h \Big ( R + t\sqrt{\frac{h}{B}} \Big ) = - C_\pm R^{m_\pm -1} \Big ( \frac{h}{BR^2}\Big )^{-\frac{1}{4}} e^{- \frac{BR^2}{4h}} \int _0^\infty e^{- \big ( \frac{s^2}{4} + ts - \sqrt{\Theta _0} s \big )} s^{\delta _0} \textrm{d}s \big (1+ o(1) \big ). \end{aligned}$$

Note that the integral is equal to $-v'(t)$. It remains to use (2.13) to find (2.9). $\square$

## Reduction when $\varvec{e_{0}}\pmb {\ne } \varvec{\frac{1}{2}}$

In all of this section, we assume that $e_0 \in \big ( - \frac{1}{2}, \frac{1}{2} \big )$, and that *h* is as small as we want along an $e_0$-sequence. In this case, Theorem 2 gives a spectral gap between the first two eigenvalues of the single-obstacle operator ${\mathscr {L}}_{h}^0$,

3.1 $$\begin{aligned} \mu _2 - \mu _1 \ge c_0 (1-2 |e_0|) h^2 + o(h^2). \end{aligned}$$

By Lemma 6, $\mu _1$ is the ground state energy of ${\mathscr {L}}_{h,m_-}$ with

$$\begin{aligned} m_- = \xi _h - e_0 + o(1) = \frac{BR^2}{2h} + \sqrt{\frac{\Theta _0 BR^2}{h}} + {\mathcal {C}}_2 - e_0 + o(1). \end{aligned}$$

The ground state eigenfunction is $\Phi ^-$.

We fix any discrete set of centers $V \subset {\mathbb {R}}^2$, with minimal distance

$$\begin{aligned} L = \inf _{\begin{array}{c} \alpha ,\beta \in V \\ \alpha \ne \beta \end{array}} | \alpha - \beta |, \end{aligned}$$

and we assume that the disks with centers $\alpha \in V$ and radius $R>0$ do not touch, i.e. $L>2R$. Then ${\mathscr {L}}_{h}^V$ denotes the magnetic-Neumann realization of ${\mathscr {L}}_{h}$ on

$$\begin{aligned} \Omega _V = {\mathbb {R}}^2{\setminus }\left( \bigcup _{\alpha \in V} D(\alpha ,R) \right) . \end{aligned}$$

We denote by $\Pi := {\textbf{1}}_{(-\infty , \mu _1 + c_0 \frac{1-2|e_0|}{2} h^2)}({\mathscr {L}}_{h}^V)$ the spectral projection of ${\mathscr {L}}_{h}^V$ corresponding to the interval $(-\infty , \mu _1 + c_0 \frac{1-2|e_0|}{2} h^2)$, and ${\mathscr {E}}_h = \mathrm{{Ran}}( \Pi )$.

### Agmon estimates

It is well known that the eigenfunctions of ${\mathscr {L}}_{h}^V$ are exponentially localized near the boundary of the disks $D(\alpha ,R)$. This follows from the so-called Agmon estimates. The following is an adaptation of the Agmon estimates in [7, Theorem 8.2.4] to states $\Psi$ which are not necessarily eigenfunctions.

#### Lemma 8

There exist $\kappa , C >0$ such that, if *h* is small enough and if $\Psi \in L^2(\Omega _V)$ satisfies $\Psi = {\textbf{1}}_{(-\infty , \frac{1+ \Theta _0}{2} B h]}({\mathscr {L}}_{h}^V)\Psi$, then

$$\begin{aligned} \int _{\Omega _V} e^{\frac{\kappa }{\sqrt{h}} {\mathfrak {d}}(x, \partial \Omega _V)} \Big ( |\Psi |^2 + h^{-1} |(-ih \nabla - A) \Psi |^2 \Big ) \textrm{d}x \le C \Vert \Psi \Vert ^2. \end{aligned}$$

Here ${\mathfrak {d}}(x,\partial \Omega _V)$ is the Euclidean distance to the obstacles, and $A=(- \frac{B}{2} x_2, \frac{B}{2} x_1)$.

#### Proof

Define for $M > 0$, the regularized (at infinity) distance

$$\begin{aligned} {\mathfrak {d}}_M =\min \{ {\mathfrak {d}},M\}. \end{aligned}$$

Let $\chi \in C^{\infty }({{\mathbb {R}}})$ be non-decreasing with $\chi (t) = 0$ for $t\le 1$, $\chi (t)=1$ for $t\ge 2$, and let $\chi _{\varepsilon }(x) = \chi ({\mathfrak {d}}/\varepsilon )$, for $\varepsilon >0$. Define

$$\begin{aligned} {{\mathcal {A}}}:= \sup _{\{\Phi = {\textbf{1}}_{(-\infty , \frac{1+ \Theta _0}{2} B h]}({\mathscr {L}}_{h}^V)\Phi \}} \frac{ \Vert \chi _{\varepsilon } e^{\frac{\kappa }{\sqrt{h}} {\mathfrak {d}}_M} \Phi \Vert }{\Vert \Phi \Vert } \le e^{\frac{\kappa }{\sqrt{h}} M}. \end{aligned}$$

We will prove that for $\varepsilon = \sqrt{h}/2$ and for sufficiently small values of *h*, ${{\mathcal {A}}}$ is bounded independently of *h* and *M*.

Let $\Psi = {\textbf{1}}_{(-\infty , \frac{1+ \Theta _0}{2} B h]}({\mathscr {L}}_{h}^V)\Psi$ be normalized. Consider the expression

3.2 $$\begin{aligned} \langle \chi _{\varepsilon } e^{\frac{\kappa }{\sqrt{h}} {\mathfrak {d}}_M} \Psi , {\mathscr {L}}_{h}^V \chi _{\varepsilon } e^{\frac{\kappa }{\sqrt{h}} {\mathfrak {d}}_M} \Psi \rangle \ge B h \Vert \chi _{\varepsilon } e^{\frac{\kappa }{\sqrt{h}} {\mathfrak {d}}_M} \Psi \Vert ^2, \end{aligned}$$

by the support properties of $\chi _{\varepsilon }$. By the so-called IMS-localization formula (or equivalently: integration by parts), we find

$$\begin{aligned} \langle \chi _{\varepsilon } e^{\frac{\kappa }{\sqrt{h}} {\mathfrak {d}}_M} \Psi ,&{\mathscr {L}}_{h}^V \chi _{\varepsilon } e^{\frac{\kappa }{\sqrt{h}} {\mathfrak {d}}_M} \Psi \rangle \\&= \Re \langle \chi _{\varepsilon }^2 e^{2\frac{\kappa }{\sqrt{h}} {\mathfrak {d}}_M} \Psi , {\mathscr {L}}_{h}^V \Psi \rangle + h^2 \langle \Psi , |\nabla (\chi _{\varepsilon } e^{\frac{\kappa }{\sqrt{h}} {\mathfrak {d}}_M} )|^2 \Psi \rangle \end{aligned}$$

We use that $\Vert \chi _\varepsilon e^{\frac{\kappa }{\sqrt{h}} {\mathfrak {d}}_M} {\mathscr {L}}_h^V \Psi \Vert \le {\mathcal {A}} \Vert {\mathscr {L}}_h^V \Psi \Vert$ to deduce

3.3 $$\begin{aligned} \langle \chi _{\varepsilon } e^{\frac{\kappa }{\sqrt{h}} {\mathfrak {d}}_M} \Psi , {\mathscr {L}}_{h}^V \chi _{\varepsilon } e^{\frac{\kappa }{\sqrt{h}} {\mathfrak {d}}_M} \Psi \rangle&\le \frac{1+ \Theta _0}{2} B h{{{\mathcal {A}}}} \Vert \chi _{\varepsilon } e^{\frac{\kappa }{\sqrt{h}} {\mathfrak {d}}_M} \Psi \Vert + 2 \kappa ^2 h\Vert \chi _{\varepsilon } e^{\frac{\kappa }{\sqrt{h}} {\mathfrak {d}}_M} \Psi \Vert ^2 \nonumber \\&\quad + 2 h^2 \varepsilon ^{-2}\Vert \chi ' \Vert _{\infty }^2 \int _{\{ 1 \le \varepsilon ^{-1} {\mathfrak {d}}(x) \le 2\}} |e^{\frac{\kappa }{\sqrt{h}} {\mathfrak {d}}_M} \Psi |^2\,dx. \end{aligned}$$

We choose $\varepsilon = \sqrt{h}/2$ and get from combining (3.2) and (3.3) and taking the supremum over all normalized $\Psi \in \textrm{Ran} {\textbf{1}}_{(-\infty , \frac{1+ \Theta _0}{2} B h]}({\mathscr {L}}_{h}^V)$,

3.4 $$\begin{aligned} Bh {{\mathcal {A}}}^2 \le (\frac{1+ \Theta _0}{2} B h + 2 h \kappa ^2) {{\mathcal {A}}}^2 + 8 h \Vert \chi ' \Vert _{\infty }^2 e^{2\kappa }. \end{aligned}$$

If $\kappa ^2 \le \frac{1- \Theta _0}{4} B$, we therefore get

3.5 $$\begin{aligned} {{\mathcal {A}}} \le \left( \frac{1- \Theta _0}{4} B - \kappa ^2\right) ^{-1} 4 \Vert \chi ' \Vert _{\infty }^2 e^{2\kappa }. \end{aligned}$$

In particular, ${{\mathcal {A}}}$ is bounded independently of *h* and *M*. Using (3.3) again, we can also bound

$$\begin{aligned} \Vert (-ih \nabla - A) \chi _\varepsilon e^{\frac{\kappa }{\sqrt{h}} {\mathfrak {d}}_M } \Psi \Vert ^2 \le Ch, \end{aligned}$$

uniformly in *M* and *h*. Therefore

$$\begin{aligned} \int _{\Omega _V} \Big ( \big | \chi _{\varepsilon } e^{\frac{\kappa }{\sqrt{h}} {\mathfrak {d}}_M } \Psi \big |^2 + h^{-1}\big | (-ih \nabla - A) \chi _{\varepsilon } e^{\frac{\kappa }{\sqrt{h}} {\mathfrak {d}}_M } \Psi \big |^2 \Big ) \textrm{d}x \le C. \end{aligned}$$

We can let $M \rightarrow \infty$ to change ${\mathfrak {d}}_M$ into ${\mathfrak {d}}$. Moreover, on the region where $\chi _\varepsilon \ne 1$ the exponential is bounded. We deduce that

$$\begin{aligned} \int _{\Omega _V} \Big ( \big | e^{\frac{\kappa }{\sqrt{h}} {\mathfrak {d}} } \Psi \big |^2 + h^{-1}\big | (-ih \nabla - A) e^{\frac{\kappa }{\sqrt{h}} {\mathfrak {d}}} \Psi \big |^2 \Big ) \textrm{d}x \le C, \end{aligned}$$

and the result follows. $\square$

In particular, using the Agmon estimates we can show that the spectrum of ${\mathscr {L}}_{h}^V$ in $(-\infty , \mu _1 + c_0 \frac{1-2|e_0|}{2} h^2)$ is exponentially close to $\mu _1$.

#### Lemma 9

If $\lambda \in \mathrm{{sp}}( {\mathscr {L}}_{h}^V)$ is such that $\lambda < \mu _1 + c_0 \frac{1-2|e_0|}{2} h^2$ then we have $|\lambda - \mu _1| \le C_N h^N$ for all $N \ge 2$, where $C_N>0$ is independent of $\lambda$ and *h*.

#### Proof

Let $\Psi$ be in the range of the spectral projector, $\Pi \Psi = \Psi$, and $(\theta _\alpha )_{\alpha \in V}$ a smooth partition of unity such that $\theta _\alpha$ is supported on a neighborhood of $D(\alpha ,R)$ and $\sum _{\alpha } \theta _\alpha ^2 =1$. Then $\theta _\alpha \Psi$ is in the domain of the single obstacle operator ${\mathscr {L}}^\alpha _h$ and by the spectral theorem

$$\begin{aligned} \sum _{\alpha \in V} \Vert ({\mathscr {L}}_{h}^\alpha - \lambda ) \theta _\alpha \Psi \Vert ^2 \ge |\mu _1 - \lambda |^2 \sum _{\alpha \in V} \Vert \theta _\alpha \Psi \Vert ^2 = |\mu _1 - \lambda |^2 \Vert \Psi \Vert ^2, \end{aligned}$$

because $\mu _1$ is the closest eigenvalue of ${\mathscr {L}}_{h}^\alpha$ to $\lambda$—this is our assumption on $\lambda$. Since $\theta _\alpha \Psi$ is also in the domain of ${\mathscr {L}}_{h}^V$,

$$\begin{aligned} \sum _{\alpha \in V} \Vert ({\mathscr {L}}_{h}^\alpha - \lambda ) \theta _\alpha \Psi \Vert ^2 \le \sum _{\alpha \in V} \Big ( \Vert \big [ {\mathscr {L}}_{h}^V, \theta _\alpha \big ] \Psi \Vert ^2 + \Vert \theta _\alpha ({\mathscr {L}}_{h}^V - \lambda ) \Psi \Vert ^2 \Big ) \end{aligned}$$

The commutator is supported away from the disks $D(\alpha ,R)$. Therefore, using Agmon estimates from Lemma 8 for $\Psi$, we deduce that it is exponentially small,

$$\begin{aligned} \sum _{\alpha \in V} \Vert ({\mathscr {L}}_{h}^\alpha - \lambda ) \theta _\alpha \Psi \Vert ^2&\le C_N h^N \Vert \Psi \Vert ^2 + \Vert ({\mathscr {L}}_{h}^V - \lambda ) \Psi \Vert ^2 \end{aligned}$$

We combine this with the lower bound to obtain

3.6 $$\begin{aligned} |\mu _1 - \lambda |^2 \le C_N h^N + \inf _{\Psi \ne 0} \frac{\Vert ( {\mathscr {L}}_h^V - \lambda ) \Pi \Psi \Vert ^2}{\Vert \Pi \Psi \Vert ^2} \end{aligned}$$

Since the operator ${\mathscr {L}}_{h}^V$ restricted to $\textrm{Ran} (\Pi )$ is selfadjoint, and $\lambda$ in its spectrum, the above infimum is vanishing. This concludes the proof. $\square$

### Approximate eigenfunctions $\Psi _\alpha$ and $g_{\alpha }$

$\Phi ^-$ is the ground state of ${\mathscr {L}}_{h}^0$. We can translate and cut-off $\Phi ^-$ to construct quasimodes for ${\mathscr {L}}_{h}^V$. For any $\eta >0$, let $\chi _\eta$ be a smooth radial cutoff function satisfying

3.7 $$\begin{aligned} \chi _\eta (x) = {\left\{ \begin{array}{ll} 1 & \quad \hbox {if}\ |x| < L-R-2 \eta \\ 0 & \quad \hbox {if}\ |x| > L-R- \eta . \end{array}\right. } \end{aligned}$$

We then define, for $\alpha \in V$,

3.8 $$\begin{aligned} \Psi _\alpha = \tau _\alpha ^B \big ( \chi _\eta \Phi ^- \big ), \qquad g_\alpha = \Pi \Psi _\alpha . \end{aligned}$$

In this section, we provide useful estimates on $g_\alpha - \Psi _\alpha$. First of all, note that the states $\Psi _\alpha$ are quasimodes for ${\mathscr {L}}_{h}^V$, approximately solving the eigenvalue equation.

#### Lemma 10

If $\eta$ is small enough then, with the function *d* defined in (2.4),

$$\begin{aligned} \Vert ({\mathscr {L}}_{h}^V - \mu _1) \Psi _\alpha \Vert = {\mathscr {O}}\big ( e^{- \frac{d(L-R-3\eta )}{h}} \big ), \quad \Vert \Psi _\alpha \Vert = 1 + {\mathscr {O}}\big ( e^{- \frac{d(L-R-3\eta )}{h}} \big ), \end{aligned}$$

and the estimates are uniform with respect to $\alpha \in V$.

#### Proof

Since $$L= \inf _{\begin{array}{c} \alpha ,\beta \in V \\ \alpha \ne \beta \end{array}} |\alpha -\beta |$$ and by definition of $\chi _\eta$, the function $\Psi _\alpha$ vanishes on an $\eta$-neighborhood of each disk $D(\beta ,R)$ for $\beta \ne \alpha$. Since it also satisfies the Neumann boundary condition on $D(\alpha ,R)$, it is in the domain of ${\mathscr {L}}_{h}^V$. Moreover $\tau _\alpha ^B$ commutes with ${\mathscr {L}}_{h}$ so we have

$$\begin{aligned} \Vert ({\mathscr {L}}_{h}^V - \mu _1) \Psi _\alpha \Vert = \Vert ({\mathscr {L}}_{h}^0 - \mu _1) \chi _\eta \Phi ^- \Vert = \Vert \big [ {\mathscr {L}}_h^0 , \chi _\eta \big ] \Phi ^- \Vert , \end{aligned}$$

where we used that $\Phi ^-$ was an eigenfunction for ${\mathscr {L}}_{h}^{0}$ with eigenvalue $\mu _1$. Now the commutator is supported at distance $|x|> L-R-2\eta$, so we can use the decay properties of $\Phi ^-$ and its gradient from Lemma 7 to get

$$\begin{aligned} \Vert ({\mathscr {L}}_{h}^V - \mu _1) \Psi _\alpha \Vert = {\mathscr {O}}\big ( e^{- \frac{d(L-R-3\eta )}{h}} \big ), \end{aligned}$$

where we replaced $2 \eta$ by $3\eta$ inside the exponential to control the front powers of $h^{-1}$. We estimate the norm similarly, using that $\Vert \tau _\alpha ^B \Phi ^- \Vert =1$ and

$$\begin{aligned} \Vert \Psi _\alpha - \tau _\alpha ^B \Phi ^- \Vert = \Vert (\chi _\eta - 1) \Phi ^{-} \Vert = {\mathscr {O}}\big ( e^{- \frac{d(L-R-3\eta )}{h}} \big ). \end{aligned}$$

Note that in these estimates the translation by $\alpha$ does not change the norm, and therefore they are independent of $\alpha$. $\square$

It follows that $g_\alpha$ is a small perturbation of $\Psi _\alpha$, and that these vectors form a basis of ${\mathscr {E}}_h$.

#### Lemma 11

For all $\alpha \in V$, the state $\Psi _\alpha$ and its projection $g_\alpha = \Pi \Psi _\alpha$ on ${\mathscr {E}}_h$ satisfy, with the function *d* defined in (2.4),

$$\begin{aligned} \Vert g_\alpha - \Psi _\alpha \Vert = {\mathscr {O}} \big ( e^{-\frac{d(L-R-4\eta )}{h}} \big ), \quad \langle {\mathscr {L}}_{h}^V (g_\alpha - \Psi _\alpha ), g_\alpha - \Psi _\alpha \rangle = {\mathscr {O}} \big ( e^{-\frac{2d(L-R-4\eta )}{h}} \big ). \end{aligned}$$

Moreover, the vectors $(g_\alpha )_{\alpha \in V}$ span a dense subspace of ${\mathscr {E}}_h$.

#### Proof

By definition of the spectral projector $\Pi$ we have

$$\begin{aligned} \Vert ({\mathscr {L}}_{h}^V - \mu _1)(I- \Pi ) \Psi _\alpha \Vert \ge c_0 \frac{(1-2|e_0|) h^2}{2} \Vert (I- \Pi ) \Psi _\alpha \Vert . \end{aligned}$$

Moreover, the projector $(I-\Pi )$ commutes with ${\mathscr {L}}_{h}^V$ so we have the upper bound

$$\begin{aligned} \Vert ({\mathscr {L}}_{h}^V - \mu _1)(I- \Pi ) \Psi _\alpha \Vert \le \Vert ({\mathscr {L}}_{h}^V - \mu _1) \Psi _\alpha \Vert = \Vert ({\mathscr {L}}_{h}^{\alpha } - \mu _1) \Psi _\alpha \Vert = {\mathscr {O}}\big ( e^{- \frac{d(L-R-3\eta )}{h}} \big ), \end{aligned}$$

where the last estimate is given by Lemma 10. Combining these two bounds we obtain

$$\begin{aligned} \Vert (I- \Pi ) \Psi _\alpha \Vert \le C h^{-2} e^{- \frac{d(L-R-3\eta )}{h}}, \end{aligned}$$

and this proves the bound on $\Vert g_\alpha - \Psi _\alpha \Vert$. To get the quadratic form bound we use a Cauchy–Schwarz inequality,

$$\begin{aligned} \langle {\mathscr {L}}_{h}^V (I - \Pi ) \Psi _\alpha , (I - \Pi ) \Psi _\alpha \rangle \le \Vert {\mathscr {L}}_{h}^V (I - \Pi ) \Psi _\alpha \Vert \Vert (I - \Pi ) \Psi _\alpha \Vert \le C h^{-4}e^{- \frac{2d(L-R-3\eta )}{h}}, \end{aligned}$$

where we used Lemma 10 again. Note that we can absorb the $h^{-4}$ by changing $3\eta$ into $4\eta$ in the exponential. The bounds are uniform with respect to $\alpha$, since the ones of Lemma 10 are.

Let us now prove that the vectors $(g_\alpha )_{\alpha \in V}$ span a dense subspace of ${\mathscr {E}}_h$. Note that, when *V* is finite $(g_\alpha )_{\alpha \in V}$ is obviously a basis for cardinality reasons, but in the general case an additional argument is needed. Assume that $\Psi \in {\mathscr {E}}_h$ is orthogonal to all $g_\alpha$’s, and we have to prove that this implies $\Psi =0$. First note that $\Psi$ is also orthogonal to $\Psi _\alpha$ since

3.9 $$\begin{aligned} \langle \Psi , \Psi _\alpha \rangle = \langle \Pi \Psi , \Psi _\alpha \rangle = \langle \Psi , g_\alpha \rangle = 0. \end{aligned}$$

We introduce a family $( \theta _\alpha )_{\alpha \in V}$ of cutoffs such that $\theta _\alpha$ is localized on a small but fixed neighborhood of $D(\alpha ,R)$, and satisfying

$$\begin{aligned} \sum _{\alpha \in V} \theta _\alpha ^2 = 1. \end{aligned}$$

We then use the IMS localization formula,

$$\begin{aligned} \langle {\mathscr {L}}_{h}^V \Psi , \Psi \rangle = \sum _{\alpha \in V} \langle {\mathscr {L}}_{h}^V \theta _\alpha \Psi , \theta _\alpha \Psi \rangle - h^2 \sum _{\alpha \in V} \Vert ( \nabla \theta _\alpha ) \Psi \Vert ^2. \end{aligned}$$

Using the Agmon estimates from Lemma 8, we know that $\Psi$ is exponentially localized near the disks. Since $\sum _\alpha |\nabla \theta _\alpha |^2$ is supported away from the disks we deduce

$$\begin{aligned} \langle {\mathscr {L}}_{h}^V \Psi , \Psi \rangle \ge \sum _{\alpha \in V} \langle {\mathscr {L}}_{h}^V \theta _\alpha \Psi , \theta _\alpha \Psi \rangle - Ch^N \Vert \Psi \Vert ^2, \end{aligned}$$

where we can choose *N* arbitrarily large (here $N=3$ is enough). Note that $\theta _\alpha \Psi$ vanishes on a neighborhood of all disks $D(\beta ,R)$ for $\beta \ne \alpha$. Therefore ${\mathscr {L}}_{h}^V \theta _\alpha \Psi = {\mathscr {L}}_{h}^\alpha \theta _\alpha \Psi$, where we recall that ${\mathscr {L}}_{h}^\alpha$ is the operator with a single obstacle $D(\alpha ,R)$, i.e. ${\mathscr {L}}_{h}^\alpha = \tau _{\alpha }^B {\mathscr {L}}_{h}^0 \tau _{-\alpha }^{B}$. Thus

3.10 $$\begin{aligned} \langle {\mathscr {L}}_{h}^V \Psi , \Psi \rangle \ge \sum _{\alpha \in V} \langle {\mathscr {L}}_{h}^0 \tau _{-\alpha }^B \theta _\alpha \Psi , \tau _{-\alpha }^B \theta _\alpha \Psi \rangle - Ch^N \Vert \Psi \Vert ^2. \end{aligned}$$

We now introduce the projection $\Pi _0$ on the ground state of ${\mathscr {L}}_{h}^0$, which is given by

$$\begin{aligned} \Pi _0 \tau _{-\alpha }^B \theta _\alpha \Psi = \langle \tau _{-\alpha }^B \theta _\alpha \Psi , \Phi ^- \rangle \Phi ^-. \end{aligned}$$

Therefore

$$\begin{aligned} \langle {\mathscr {L}}_{h}^0 \tau _{-\alpha }^B \theta _\alpha \Psi , \tau _{-\alpha }^B \chi _\alpha \Psi \rangle&= \mu _1 | \langle \tau _{-\alpha }^B \theta _\alpha \Psi , \Phi ^- \rangle |^2 + \langle {\mathscr {L}}_{h}^0 (I-\Pi _0) \tau _{-\alpha }^B \theta _\alpha \Psi , (I-\Pi _0) \tau _{-\alpha }^B \theta _\alpha \Psi \rangle \\&\ge \mu _1 | \langle \tau _{-\alpha }^B \theta _\alpha \Psi , \Phi ^- \rangle |^2 + (\mu _1 + c_0(1-2|e_0|) h^2) \Vert (I-\Pi _0) \tau _{-\alpha }^B \theta _\alpha \Psi \Vert ^2, \end{aligned}$$

where we used that the second eigenvalue of ${\mathscr {L}}_{h}^0$ has a gap of size $c_0 (1-2|e_0|) h^2$ with $\mu _1$ (Theorem 2). We reconstruct the $L^2$ norm of $\tau _{-\alpha }^B \theta _\alpha \Psi$ and use that $\tau _{-\alpha }^B$ is an isometry to get

3.11 $$\begin{aligned}&\langle {\mathscr {L}}_{h}^0 \tau _{-\alpha }^B \theta _\alpha \Psi , \tau _{-\alpha }^B \chi _\alpha \Psi \rangle \nonumber \\&\ge \big ( \mu _1 + c_0(1-2|e_0|) h^2 \big ) \Vert \theta _\alpha \Psi \Vert ^2 - c_0(1-2|e_0|) h^2 | \langle \tau _{-\alpha }^B \theta _\alpha \Psi , \Phi ^- \rangle |^2. \end{aligned}$$

The last term can be estimated as follows. First introduce the cutoff $\chi _\eta$ defining $\Psi _\alpha$,

$$\begin{aligned} \langle \tau _{-\alpha }^B \theta _\alpha \Psi , \Phi ^- \rangle = \langle \theta _\alpha \Psi , \tau _\alpha ^B \chi _\eta \Phi ^- \rangle + \langle \theta _\alpha \Psi , \tau _\alpha ^B (1-\chi _\eta ) \Phi ^- \rangle . \end{aligned}$$

Using the decay of $\Phi ^-$ and the cutoff we see that the second term is exponentially small. In the first one we use the orthogonality $\langle \Psi , \Psi _\alpha \rangle =0$ and we find

$$\begin{aligned} \langle \tau _{-\alpha }^B \theta _\alpha \Psi , \Phi ^- \rangle&\le \langle (\theta _\alpha -1) \Psi , \tau _\alpha ^B \chi _\eta \Phi ^- \rangle +Ch^N \Vert \theta _\alpha \Psi \Vert \\&\le \Vert \chi _\eta ( . -\alpha ) \big ( \theta _\alpha -1 \big ) \Psi \Vert +Ch^N \Vert \theta _\alpha \Psi \Vert . \end{aligned}$$

We insert this last inequality in (3.11) and equation (3.10) now reads

3.12 $$\begin{aligned} \langle {\mathscr {L}}_{h}^V \Psi , \Psi \rangle \ge \big ( \mu _1 + c_0(1-2|e_0|) h^2 - Ch^N \big ) \Vert \Psi \Vert ^2 - C h^2 \sum _{\alpha \in V} \Vert \chi _\eta (. -\alpha ) \big ( \theta _\alpha -1 \big ) \Psi \Vert ^2.\nonumber \\ \end{aligned}$$

Since the function $\sum _\alpha \chi _\eta (.-\alpha )^2 \big ( \theta _\alpha -1 \big )^2$ is supported away from the obstacles, we can use Agmon estimates again to see that the last term is exponentially small, and therefore

3.13 $$\begin{aligned} \langle {\mathscr {L}}_{h}^V \Psi , \Psi \rangle \ge \big ( \mu _1 + c_0(1-2|e_0|) h^2 - Ch^N \big ) \Vert \Psi \Vert ^2. \end{aligned}$$

But $\Psi \in {\mathscr {E}}_h$, meaning that $\langle {\mathscr {L}}_{h}^V \Psi , \Psi \rangle \le ( \mu _1 + c_0 \frac{1-2|e_0|}{2}h^2) \Vert \Psi \Vert ^2$, and therefore we must have $\Psi = 0$. This proves that $(g_\alpha )$ generates a dense subspace of ${\mathscr {E}}_h$. $\square$

In the case when the set of disks *V* is infinite, we need a little more information on the decay of $g_\alpha - \Psi _\alpha$, which we give in the following lemma. This result is very weak, since we only get decay when $\langle x - \alpha \rangle \gg h^{-1}$. However, this is enough to obtain decay of coefficients of our infinite interaction matrix away from the diagonal, in the proof of Theorem 13.

#### Lemma 12

For all $\alpha \in V$ we have

$$\begin{aligned} \Vert e^{ h \langle x - \alpha \rangle } ( g_\alpha - \Psi _\alpha ) \Vert = {\mathscr {O}}( e^{-\frac{d(L-R-4\eta )}{h}} ), \qquad \Vert e^{ h \langle x - \alpha \rangle } {\mathscr {L}}_{h}^V ( g_\alpha - \Psi _\alpha ) \Vert = {\mathscr {O}}( e^{-\frac{d(L-R-4\eta )}{h}} ), \end{aligned}$$

where $$\langle x \rangle = (1+ |x|^2)^{1/2}$$.

#### Proof

We recall that $\Psi _\alpha$, defined in (3.8), is an approximate eigenfunction for ${\mathscr {L}}_{h}^V$. We define the remainder

$$\begin{aligned} r_\alpha = ({\mathscr {L}}_{h}^V - \mu _1) \Psi _\alpha , \end{aligned}$$

which satisfies

3.14 $$\begin{aligned} ({\mathscr {L}}_{h}^V - z)^{-1} \Psi _\alpha = \frac{1}{\mu _1 - z} \Psi _\alpha - \frac{1}{\mu _1-z} ({\mathscr {L}}_{h}^V-z)^{-1} r_{\alpha }, \quad \forall z \notin \mathrm{{sp}}( {\mathscr {L}}_{h}^V). \end{aligned}$$

Also note that the spectral projection $g_\alpha =\Pi \Psi _\alpha$ can be written as the path-integral

3.15 $$\begin{aligned} g_\alpha = - \frac{1}{2\pi i} \int _{\Gamma } ({\mathscr {L}}_{h}^V - z)^{-1} \Psi _\alpha \textrm{d}z, \end{aligned}$$

where $\Gamma$ is a (clock-wise) circle of center $\mu _1$ and radius $c_0 \frac{1-2|e_0|}{2}h^2$. Therefore using (3.14), we have

3.16 $$\begin{aligned} g_\alpha - \Psi _\alpha = \frac{1}{2\pi i} \int _{\Gamma } \frac{1}{\mu _1 -z} ({\mathscr {L}}_{h}^V - z)^{-1} r_\alpha \textrm{d}z, \end{aligned}$$

and

3.17 $$\begin{aligned} \Vert e^{ h \langle x- \alpha \rangle } (g_\alpha - \Psi _\alpha ) \Vert \le C \sup _{z \in \Gamma } \Vert e^{ h \langle x-\alpha \rangle } ({\mathscr {L}}_{h}^V - z)^{-1} r_\alpha \Vert \end{aligned}$$

In order to bound the right-hand side, we first consider the weight $\phi (x) = \langle x - \alpha \rangle \rho (x/M)$, where $\rho \in C^\infty _0({\mathbb {R}}^2)$ is equal to 1 on *D*(0, 1). We will prove estimates uniform in $M\ge 1$ and then let $M \rightarrow \infty$.

For $z \in \Gamma$, we use the resolvent commutation formula to find

3.18 $$\begin{aligned} \Vert e^{ h \phi } ({\mathscr {L}}_{h}^V - z)^{-1} r_\alpha \Vert&\le \Vert ({\mathscr {L}}_{h}^V-z)^{-1} e^{ h \phi } r_\alpha \Vert + \Vert ({\mathscr {L}}_{h}^V -z)^{-1} \big [ {\mathscr {L}}_{h}^V, e^{ h \phi }\big ] ({\mathscr {L}}_{h}^V - z)^{-1} r_\alpha \Vert \nonumber \\&\le C h^{-2} \Vert e^{ h \phi } r_\alpha \Vert + C h^{-2} \Vert \big [ {\mathscr {L}}_{h}^V, e^{ h \phi }\big ] ({\mathscr {L}}_{h}^V - z)^{-1} r_\alpha \Vert , \end{aligned}$$

where we used the spectral theorem and Lemma 9 to bound the norm of the resolvent. Let us now investigate the commutator, which is explicitly given by

$$\begin{aligned} \big [ {\mathscr {L}}_{h}^V, e^{ h \phi } \big ] = -e^{ h \phi }\big ( 2i h^2 \nabla \phi \cdot (-ih\nabla - A) + h^3 \Delta \phi + h^4 |\nabla \phi |^2 \big ), \end{aligned}$$

where $A=(-\frac{B}{2} x_2, \frac{B}{2} x_1)$ is the vector potential. Since $\nabla \phi$ and $\Delta \phi$ are bounded (uniformly with respect to $\alpha$) we deduce

3.19 $$\begin{aligned} \Vert \big [ {\mathscr {L}}_{h}^V, e^{ h \phi } \big ] ({\mathscr {L}}_{h}^V - z)^{-1} r_\alpha \Vert&\le C h^2 \Vert e^{ h \phi } (-ih\nabla - A) ({\mathscr {L}}_{h}-z)^{-1} r_\alpha \Vert \nonumber \\&\qquad + C h^3 \Vert e^{ h \phi } ({\mathscr {L}}_{h}-z)^{-1} r_\alpha \Vert . \end{aligned}$$

The first term in (3.19) we bound as follows using the notation $u = ({\mathscr {L}}_{h}^V - z)^{-1} r_\alpha$. We integrate by parts, and since $u \in \mathrm{{Dom}}({\mathscr {L}}_{h}^V)$ has Neumann boundary conditions, we find

$$\begin{aligned} \Vert e^{ h \phi } (-ih \nabla - A) u \Vert ^2&= \langle (-ih \nabla - A) e^{2 h \phi } (-ih\nabla - A) u, u \rangle \\&\le | \langle e^{2 h \phi } {\mathscr {L}}_{h}u, u \rangle | + 2| \langle h^2 \nabla \phi e^{ h \phi } (-ih\nabla - A)u,e^{ h \phi } u \rangle |\\&\le \Vert e^{ h \phi } {\mathscr {L}}_{h}u \Vert \Vert e^{ h \phi } u \Vert + C h^2 \Vert e^{ h \phi } (-ih\nabla - A)u \Vert \Vert e^{ h \phi } u \Vert . \end{aligned}$$

In the last term, we use the Cauchy inequality and compare with the left-hand side to get

$$\begin{aligned} \Vert e^{ h \phi } (-ih \nabla - A) u \Vert ^2&\le C \Vert e^{ h \phi } {\mathscr {L}}_{h}u \Vert \Vert e^{ h \phi } u \Vert + C h^4\Vert e^{ h \phi } u \Vert ^2 \\&\le C\Vert e^{ h \phi } ({\mathscr {L}}_{h}^V - z) u \Vert \Vert e^{ h \phi } u \Vert + C h \Vert e^{ h \phi } u \Vert ^2\\&\le C h^{-1} \Vert e^{ h \phi } ({\mathscr {L}}_{h}^V - z) u \Vert ^2 + C h \Vert e^{ h \phi } u \Vert ^2 . \end{aligned}$$

We recall that $u = ({\mathscr {L}}_{h}^V - z)^{-1} r_\alpha$ and insert this last bound in (3.19),

$$\begin{aligned} \Vert \big [ {\mathscr {L}}_{h}^V, e^{ h \phi } \big ] ({\mathscr {L}}_{h}^V - z)^{-1} r_\alpha \Vert&\le C h^{3/2} \Vert e^{ h \phi } r_\alpha \Vert + C h^{5/2} \Vert e^{ h \phi } ({\mathscr {L}}_{h}-z)^{-1} r_\alpha \Vert . \end{aligned}$$

We now insert in (3.18) and obtain

$$\begin{aligned} \Vert e^{ h \phi } ({\mathscr {L}}_{h}^V - z)^{-1} r_\alpha \Vert \le C h^{-2} \Vert e^{ h \phi } r_\alpha \Vert + C h^{1/2} \Vert e^{ h \phi } ({\mathscr {L}}_{h}^V - z)^{-1} r_\alpha \Vert . \end{aligned}$$

We substract the last term from the left-hand side and get

$$\begin{aligned} \Vert e^{ h \phi } ({\mathscr {L}}_{h}^V - z)^{-1} r_\alpha \Vert \le C h^{-2} \Vert e^{ h \phi } r_\alpha \Vert . \end{aligned}$$

Then, we let $M \rightarrow \infty$, so that $\phi \rightarrow \langle x-\alpha \rangle$, and with (3.17) this gives

$$\begin{aligned} \Vert e^{ h \langle x- \alpha \rangle } (g_\alpha - \Psi _\alpha ) \Vert \le Ch^{-2} \Vert e^{ h \langle x-\alpha \rangle } r_\alpha \Vert . \end{aligned}$$

Finally, we can estimate the norm of $e^{ h\langle x-\alpha \rangle } r_\alpha$ as in Lemma 10. Indeed, this estimate follows from the decay of the groundstate $\Phi ^-$ of ${\mathscr {L}}_{h}^0$, which is much faster than $e^{h\langle x-\alpha \rangle }$, and the proof is thus identical. We obtain the first stated result. For the second one, we use (3.16) again and apply ${\mathscr {L}}_{h}^V$ to find

$$\begin{aligned} \Vert e^{ h \langle x - \alpha \rangle } {\mathscr {L}}_{h}^V ( g_\alpha - \Psi _\alpha ) \Vert \le C \Vert e^{h \langle x - \alpha \rangle } r_\alpha \Vert + C \sup _{z \in \Gamma } |z| \Vert e^{h \langle x - \alpha \rangle } ({\mathscr {L}}_{h}^V - z)^{-1} r_\alpha \Vert , \end{aligned}$$

and these two terms we estimate as above. $\square$

### The Gram matrix

In the previous section, we constructed a basis $(g_\alpha )_{\alpha \in V}$ of the spectral subspace ${\mathscr {E}}_h$. Before estimating the spectrum of ${\mathscr {L}}_{h}^V$ restricted to ${\mathscr {E}}_h$, we orthonormalize the basis $(g_\alpha )_{\alpha \in V}$, thus providing a Hilbertian basis $(e_\alpha )_{\alpha \in V}$ of ${\mathscr {E}}_h$. This is done using the Gram matrix ${\textbf{G}}$. For any $\varepsilon >0$ we define the error order

3.20 $$\begin{aligned} {\mathcal {E}}_{\varepsilon ,L} = e^{- \frac{2S_h(L)}{h}} + e^{-\frac{2d(L-R-4\eta )}{h}}+ e^{-\frac{S_h(L(1+\varepsilon ))}{h}} + e^{-\frac{d(L-R-4\eta ) + d(\varepsilon L + R + 2 \eta )}{h}}, \end{aligned}$$

where $S_h$ was defined in (1.3). This error is shown to be small enough in Lemma 21.

#### Theorem 13

Let $\varepsilon >0$ and assume $h>0$ is small enough along an $e_0$-sequence with $e_0 \in (-\frac{1}{2}, \frac{1}{2})$. For all $p \in [1, \infty ]$, the infinite matrix ${\textbf{G}}= (\langle g_\alpha , g_\beta \rangle )_{\alpha , \beta \in V}$ defines a bounded operator on $\ell ^p(V)$. Moreover,

1. We have the following estimate in $\ell ^p$ operator norm topology
  $$\begin{aligned} {\textbf{G}}= {\textbf{I}} + {\textbf{T}}+ {\mathscr {O}}({\mathcal {E}}_{\varepsilon ,L}), \quad {\text {in}}\quad {\mathcal {L}}(\ell ^p(V)), \end{aligned}$$
  where $${\textbf{T}}_{\alpha \beta } = \langle g_\alpha , g_\beta \rangle {\textbf{1}}_{L \le |\alpha - \beta | \le L(1 + \varepsilon )}$$.
2. ${\textbf{G}}^{-1}$ is a bounded operator on $\ell ^p(V)$ and has a square root which satisfies
  $$\begin{aligned} {\textbf{G}}^{- \frac{1}{2}} = {\textbf{I}} - \frac{1}{2} {\textbf{T}}+ {\mathscr {O}}({\mathcal {E}}_{\varepsilon ,L}), \quad {\text {in}}\quad {\mathcal {L}}(\ell ^p(V)). \end{aligned}$$
3. The vectors
  $$\begin{aligned} e_\alpha = \sum _{\beta \in V} ({\textbf{G}}^{- \frac{1}{2}})_{\alpha \beta } g_\beta , \qquad \alpha \in V, \end{aligned}$$
  define a Hilbert basis of ${\mathscr {E}}_h$.
4. The restriction of ${\mathscr {L}}_{h}^V$ to ${\mathscr {E}}_h$ is unitarily equivalent to ${\textbf{M}}= ( \langle {\mathscr {L}}_{h}^V e_\alpha , e_\beta \rangle )_{\alpha , \beta \in V}$ as an operator on $\ell ^2(V)$, and
  $$\begin{aligned} {\textbf{M}}= \mu _1 {\textbf{I}} + {\textbf{W}} +{\mathscr {O}}({\mathcal {E}}_{\varepsilon ,L}), \end{aligned}$$
  where $${\textbf{W}}_{\alpha \beta } = \langle ({\mathscr {L}}_{h}- \mu _1) \Psi _\alpha , \Psi _\beta \rangle {\textbf{1}}_{L \le |\alpha - \beta | \le L(1 + \varepsilon )}$$.

#### Proof

Some of the technical estimates on the interaction coefficients required in the proof of Theorem 13 are postponed to Appendix B below.

1. We prove the result for $p=1$ and $p=\infty$. The general statement then follows by the interpolation inequality,

$$\begin{aligned} \Vert {\textbf{A}} \Vert _p \le \Vert {\textbf{A}} \Vert _1^{\frac{1}{p}} \Vert {\textbf{A}} \Vert _{\infty }^{1-\frac{1}{p}}, \end{aligned}$$

where here $\Vert {\textbf{A}} \Vert _p$ is the operator norm $\ell ^p(V) \rightarrow \ell ^p(V)$. Note that for hermitian matrices like ${\textbf{G}}$, the $\ell ^1$-norm and $\ell ^{\infty }$-norm are equal and given by

$$\begin{aligned} \Vert {\textbf{A}} \Vert _1 = \Vert {\textbf{A}} \Vert _{\infty } = \sup _{\alpha \in V} \sum _{\beta \in V} |{\textbf{A}}_{\alpha \beta }|. \end{aligned}$$

We can write ${\textbf{G}}$ as follows, decomposing according to $|\alpha - \beta |$,

$$\begin{aligned} {\textbf{G}}= \widetilde{{\textbf{I}}} + {\textbf{T}} + \widetilde{{\textbf{R}}}, \end{aligned}$$

where

$$\begin{aligned} \widetilde{{\textbf{I}}}_{\alpha \beta } = \Vert g_\alpha \Vert ^2 {\textbf{1}}_{\alpha = \beta }, \quad {{\textbf{T}}}_{\alpha \beta } = \langle g_\alpha , g_\beta \rangle {\textbf{1}}_{L \le |\alpha - \beta | \le L(1 + \varepsilon )}, \quad \widetilde{{\textbf{R}}}_{\alpha \beta } = \langle g_\alpha , g_\beta \rangle {\textbf{1}}_{|\alpha - \beta | >L(1 + \varepsilon )}. \end{aligned}$$

We first estimate the norm of $\widetilde{{\textbf{R}}}$,

$$\begin{aligned} \Vert \widetilde{{\textbf{R}}} \Vert _1 = \sup _{\alpha \in V} \sum _{\begin{array}{c} \beta \in V \\ |\alpha - \beta |>L(1 + \varepsilon ) \end{array}} |\langle g_\alpha , g_\beta \rangle |. \end{aligned}$$

By the Pythagorean Theorem we have $\langle g_\alpha , g_\beta \rangle = \langle \Psi _\alpha , \Psi _\beta \rangle + \langle g_\alpha - \Psi _\alpha , g_\beta - \Psi _\beta \rangle$. Remember that $\Psi _\alpha$ is supported on $D(\alpha , L-R-\eta )$. Therefore, given $\alpha \in V$, the scalar product $\langle \Psi _\alpha , \Psi _\beta \rangle$ vanishes for all but finitely many $\beta$’s. For those we can use Lemma 18 below, and we deduce

3.21 $$\begin{aligned} \Vert \widetilde{{\textbf{R}}} \Vert _1 \le \sup _{\alpha \in V} \sum _{\begin{array}{c} \beta \in V \\ |\alpha - \beta |>L(1 + \varepsilon ) \end{array}} | \langle g_\alpha - \Psi _\alpha , g_\beta - \Psi _\beta \rangle | + {\mathscr {O}} \big (e^{-\frac{S_h(L(1+ \varepsilon ))}{h}} + e^{-\frac{d(L-R-3\eta ) + d(\varepsilon L + R + 2 \eta )}{h}} \big ) \end{aligned}$$

To estimate the remaining sum, we use that $\langle \alpha - \beta \rangle \le \langle \alpha - x \rangle + \langle \beta - x \rangle$ to insert a decaying weight,

$$\begin{aligned} | \langle g_\alpha - \Psi _\alpha , g_\beta - \Psi _\beta \rangle | \le e^{-h \langle \alpha - \beta \rangle } \Vert e^{h \langle x - \alpha \rangle }( g_\alpha - \Psi _\alpha ) \Vert \Vert e^{h \langle x - \beta \rangle }( g_\beta - \Psi _\beta ) \Vert , \end{aligned}$$

and then we use Lemma 12 to get

$$\begin{aligned} | \langle g_\alpha - \Psi _\alpha , g_\beta - \Psi _\beta \rangle | \le C e^{-h\langle \alpha - \beta \rangle } e^{- \frac{2 d(L-R-4\eta )}{h}}. \end{aligned}$$

The sum over $\beta$ is convergent and (3.21) becomes

3.22 $$\begin{aligned} \Vert \widetilde{{\textbf{R}} }\Vert _1 \le C \big (e^{- \frac{2 d(L-R-4\eta )}{h}} + e^{-\frac{S_h(L(1+\varepsilon ))}{h}} + e^{-\frac{d(L-R-4\eta ) + d(\varepsilon L + R + 2 \eta )}{h}} \big ). \end{aligned}$$

Similarly we can estimate

$$\begin{aligned} \Vert {\textbf{I}} - \widetilde{{\textbf{I}}} \Vert _1&\le C \sup _{\alpha \in V} | \Vert g_\alpha \Vert ^2 -1 | \le C e^{- \frac{2d(L-R- 4\eta )}{h}}, \end{aligned}$$

using Lemma 11. This proves the first item.

2. Note that the previous discussion also gives, for $p \in [1,\infty ]$,

$$\begin{aligned} \Vert {\textbf{G}} - {\textbf{I}} \Vert _p \le \Vert {\textbf{T}} \Vert _p + {\mathscr {O}}(e^{- \frac{2 d(L-R-4\eta )}{h}} + e^{-\frac{S_h(L(1+\varepsilon ))}{h}} + e^{-\frac{d(L-R-4\eta ) + d(\varepsilon L + R + 2 \eta )}{h}}), \end{aligned}$$

and using Lemma 11, as well as Lemma 18 from Appendix B, we get

$$\begin{aligned} \Vert {\textbf{T}} \Vert _p \le \Vert {\textbf{T}} \Vert _1&\le C \sup _{L \le |\alpha - \beta | \le L(1 + \varepsilon )} | \langle \Psi _\alpha , \Psi _\beta \rangle | + C e^{-\frac{2d(L-R-4\eta )}{h}} \\  &\le C \big (e^{-\frac{S_h(L)}{h}} + e^{-\frac{d(L-R-3\eta )}{h}} \big ). \end{aligned}$$

Therefore ${\textbf{G}}$ is a perturbation of the identity, and as such it is invertible. Moreover, ${\textbf{G}}^{-1/2}$ exists—as sum of a convergent Taylor series—and the first terms of the series give

$$\begin{aligned} \Vert {\textbf{G}}^{- 1/2} - {\textbf{I}} + \frac{1}{2} {\textbf{T}} \Vert _p \le C \Vert {\textbf{T}}\Vert _p^2 + C \big (e^{- \frac{2 d(L-R-4\eta )}{h}} + e^{-\frac{S_h(L(1+\varepsilon ))}{h}} + e^{-\frac{d(L-R-4\eta ) + d(\varepsilon L + R + 2 \eta )}{h}} \big ), \end{aligned}$$

which proves the second item.

3. The series defining $e_\alpha$ is convergent in ${\mathscr {E}}_h$ because ${\textbf{G}}^{- \frac{1}{2}}$ is a bounded operator on $\ell ^{1}(V)$ and

$$\begin{aligned} \sum _{\beta \in V} | ({\textbf{G}}^{-\frac{1}{2}})_{\alpha \beta }| \Vert g_\beta \Vert \le C \sum _{\beta } | ({\textbf{G}}^{-\frac{1}{2}})_{\alpha \beta }| \le C \Vert {\textbf{G}} \Vert _1. \end{aligned}$$

Moreover, item (2) for $p=1$ yields

3.23 $$\begin{aligned} e_\alpha = g_\alpha - \frac{1}{2} \sum _{\begin{array}{c} \beta \in V \\ L \le |\alpha - \beta |\le L(1 + \varepsilon ) \end{array}} \langle g_\alpha , g_\beta \rangle g_\beta + {\mathscr {O}}({\mathcal {E}}_{\varepsilon , L}), \end{aligned}$$

uniformly with respect ot $\alpha$. In particular, $e_\alpha$ is a perturbation of $g_\alpha$. Using Lemma 11, we deduce that it spans a dense subspace of ${\mathscr {E}}_h$. It is also an orthonormal family, since

$$\begin{aligned} \langle e_\alpha , e_{\alpha '} \rangle&= \sum _{\beta , \beta ' \in V} {\textbf{G}}^{- \frac{1}{2}}_{\alpha \beta } \langle g_\beta , g_{\beta '} \rangle \overline{{\textbf{G}}^{- \frac{1}{2}}_{\alpha ' \beta '}} = ({\textbf{G}}^{- \frac{1}{2}} {\textbf{G}}{\textbf{G}}^{- \frac{1}{2}})_{\alpha \alpha '} = {\textbf{I}}_{\alpha \alpha '}. \end{aligned}$$

4. Since $(e_\alpha )_{\alpha \in V}$ is a Hilbert basis of ${\mathscr {E}}_h$, the operator ${\mathscr {L}}_{h}$ restricted to ${\mathscr {E}}_h$ is obviously unitary equivalent to ${\textbf{M}}$, the unitary being

$$\begin{aligned}{\textbf{U}}: x \in \ell ^2(V) \mapsto \sum _{\alpha } x_\alpha e_{\alpha } \in {\mathscr {E}}_h. \end{aligned}$$

To obtain the estimates on ${\textbf{M}}$, let us first introduce the infinite matrix

$$\begin{aligned} {\widetilde{{\textbf{M}}}}= ( \langle {\mathscr {L}}_{h}^V g_\alpha , g_\beta \rangle )_{\alpha , \beta \in V}. \end{aligned}$$

We claim that ${\widetilde{{\textbf{M}}}}$ defines a bounded operator on $\ell ^1(V)$ such that

3.24 $$\begin{aligned} {\widetilde{{\textbf{M}}}} = \mu _1 {\textbf{I}} + {\textbf{W}} + \mu _1 {\textbf{T}} + {\mathscr {O}}({\mathcal {E}}_{\varepsilon ,L}), \end{aligned}$$

in $\ell ^1$ operator norm topology. Indeed, for $\alpha = \beta$ we have

$$\begin{aligned} \langle {\mathscr {L}}_{h}^V g_\alpha , g_\alpha \rangle&= \mu _1 \Vert g_\alpha \Vert ^2 + \langle ({\mathscr {L}}_{h}^V - \mu _1) g_\alpha , g_\alpha \rangle \\&= \mu _1 \Vert g_\alpha \Vert ^2 + \langle ({\mathscr {L}}_{h}^V - \mu _1) \Psi _\alpha , \Psi _\alpha \rangle + \langle ({\mathscr {L}}_{h}^V - \mu _1) ( g_\alpha - \Psi _\alpha ), ( g_\alpha - \Psi _\alpha ) \rangle \\&= \mu _1 + {\mathscr {O}}(e^{- \frac{2d(L-R-4\eta )}{h}}), \end{aligned}$$

by Lemmas 10 and 11. For $L \le |\alpha - \beta | \le L(1 + \varepsilon )$ we have

$$\begin{aligned} \langle {\mathscr {L}}_{h}^V g_\alpha , g_\beta \rangle {\textbf{1}}_{L \le |\alpha - \beta | \le L(1 + \varepsilon )}&= \Big ( \langle ({\mathscr {L}}_{h}^V - \mu _1) g_\alpha , g_\beta \rangle + \mu _1 \langle g_\alpha , g_\beta \rangle \Big ) {\textbf{1}}_{L \le |\alpha - \beta | \le L(1 + \varepsilon )}\\  &={\textbf{W}}_{\alpha \beta } + \mu _1 {\textbf{T}}_{\alpha \beta } + {\mathscr {O}}(e^{- \frac{2d(L-R-4\eta )}{h}}), \end{aligned}$$

where we used Lemma 11 again. Finally the last part we estimate as before,

$$\begin{aligned}&\sup _{\alpha \in V} \sum _{\begin{array}{c} \beta \in V \\ | \alpha - \beta |>L(1 + \varepsilon ) \end{array}} |\langle {\mathscr {L}}_{h}^V g_\alpha , g_\beta \rangle | \\&\quad \le \sup _{\alpha \in V} \sum _{\begin{array}{c} \beta \in V | \alpha - \beta | >L(1 + \varepsilon ) \end{array}} |\langle {\mathscr {L}}_{h}^V (g_\alpha - \Psi _\alpha ), g_\beta - \Psi _\beta \rangle | + |\langle {\mathscr {L}}_{h}^V \Psi _\alpha , \Psi _\beta \rangle |. \end{aligned}$$

The second term vanishes for all but finitely many $\beta$’s. For the remaining ones we use Lemma 20. In the first term we insert the exponential weight and find

$$\begin{aligned}&\sup _{\alpha \in V} \sum _{\begin{array}{c} \beta \in V \\ | \alpha - \beta |>L(1 + \varepsilon ) \end{array}} |\langle {\mathscr {L}}_{h}^V g_\alpha , g_\beta \rangle | \\  &\qquad \le \sup _{\alpha \in V} \sum _{\begin{array}{c} \beta \in V \\ | \alpha - \beta | >L(1 + \varepsilon ) \end{array}} e^{-h \langle \alpha - \beta \rangle } \Vert e^{h \langle x - \alpha \rangle } {\mathscr {L}}_{h}^V (g_\alpha - \Psi _\alpha ) \Vert \Vert e^{h \langle x - \beta \rangle } ( g_\beta - \Psi _\beta ) \Vert \\  &\qquad \qquad + {\mathscr {O}}(e^{- \frac{S_h(L(1+\varepsilon ))}{h}}+ e^{-\frac{d(L-R- 3 \eta ) + d(\varepsilon L + R + 2 \eta )}{h}}+e^{-\frac{2d(L-R-4\eta )}{h}}). \end{aligned}$$

We then use Lemma 12 to obtain

$$\begin{aligned} \sup _{\alpha \in V} \sum _{\begin{array}{c} \beta \in V \\ | \alpha - \beta | >L(1 + \varepsilon ) \end{array}} |\langle {\mathscr {L}}_{h}^V g_\alpha , g_\beta \rangle | \le {\mathscr {O}}(e^{- \frac{S_h(L(1+\varepsilon ))}{h}}+ e^{-\frac{d(L-R- 3 \eta ) + d(\varepsilon L + R + 2 \eta )}{h}}+e^{-\frac{2d(L-R-4\eta )}{h}}). \end{aligned}$$

and this proves (3.24).

By definition of ${\textbf{M}}$ and $e_{\alpha }$ we have

$$\begin{aligned} {\textbf{M}}_{\alpha \alpha '} = \sum _{\beta \beta ' \in V} {\textbf{G}}^{- \frac{1}{2}}_{\alpha \beta } {\textbf{G}}^{- \frac{1}{2}}_{\alpha ' \beta '} {\widetilde{{\textbf{M}}}}_{\beta \beta '} = ({\textbf{G}}^{- \frac{1}{2}} {\widetilde{{\textbf{M}}}} {\textbf{G}}^{- \frac{1}{2}} )_{\alpha \alpha '}. \end{aligned}$$

Moreover using item (2) and (3.24) we have

$$\begin{aligned} {\textbf{G}}^{- \frac{1}{2}} {\widetilde{{\textbf{M}}}} {\textbf{G}}^{- \frac{1}{2}} = \mu _1 {\textbf{I}} + {\textbf{W}} + {\mathscr {O}}({\mathcal {E}}_{\varepsilon ,L}), \end{aligned}$$

in $\ell ^1$ operator norm topology. We deduce that the remainder ${\textbf{S}} = {\textbf{M}}- \mu _1 {\textbf{I}} - {\textbf{W}}$ is small enough in $\ell ^1$ operator norm topology, but also in $\ell ^{\infty }$ norm topology since it is symmetric. Using interpolation we deduce

$$\begin{aligned} {\textbf{M}}- \mu _1 {\textbf{I}} - {\textbf{W}} = {\mathscr {O}}({\mathcal {E}}_{\varepsilon ,L}) \end{aligned}$$

in $\ell ^2$ operator norm topology. $\square$

## Reduction When $\varvec{e_0} \pmb {=} \varvec{\frac{1}{2}}$

We now consider the case $e_0= \frac{1}{2}$, and we assume in all of this section that *h* is as small as we want along a $\frac{1}{2}$-sequence. In this case, the first eigenvalue of the single-obstacle operator ${\mathscr {L}}_{h}^0$ might be double. However, Theorem 2 ensures that there is a gap with the third eigenvalue,

4.1 $$\begin{aligned} \mu _3 - \mu _2 \ge 2c_0 h^2 + o(h^2). \end{aligned}$$

By Lemma 6, the first two eigenvalues are $\lbrace \mu _-,\mu _+ \rbrace$ where $\mu _\pm$ is the ground state energy of ${\mathscr {L}}_{h,m_\pm }$ and

4.2 $$\begin{aligned} m_-&= \xi _h - \frac{1}{2} + o(1) = \frac{BR^2}{2h} + \sqrt{\frac{\Theta _0 BR^2}{h}} + {\mathcal {C}}_2 - \frac{1}{2} + o(1), \end{aligned}$$

4.3 $$\begin{aligned} m_+&= \xi _h + \frac{1}{2} + o(1)= \frac{BR^2}{2h} + \sqrt{\frac{\Theta _0 BR^2}{h}} + {\mathcal {C}}_2 + \frac{1}{2} + o(1). \end{aligned}$$

Depending on *h*, either $\mu _-$ or $\mu _+$ can be the smallest. The associated eigenfunctions are $\Phi ^-$ and $\Phi ^+$. We denote by $\Pi$ the spectral projector of ${\mathscr {L}}_{h}^V$ corresponding to the interval $(-\infty , \mu _2 +c_0 h^2)$.

The reduction in this case follows essentially the same lines as in Sect. 3, except that the eigenspace of the single-obstacle operator is now bidimensional. Using Agmon estimates (Lemma 8), we know that the spectrum of ${\mathscr {L}}_{h}^V$ is exponentially close to $\mu _1$.

### Lemma 14

If $\lambda \in \mathrm{{sp}}({\mathscr {L}}_{h}^V)$ is such that $\lambda < \mu _2 +c_0 h^2$ then we have

$$\begin{aligned} \min \big ( |\lambda - \mu _-|, |\lambda - \mu _+| \big ) \le C_N h^N \end{aligned}$$

for all $N \ge 2$, where $C_N>0$ is independent of $\lambda$ and *h*.

### Proof

The proof is identical to Lemma 9, up to changing the distance $|\lambda - \mu _1|$ by the minimal distance $\min \big ( |\lambda - \mu _-|, |\lambda - \mu _+| \big )$. $\square$

Recalling the definition (3.7) of the cutoff function $\chi _\eta$, we define for $\alpha \in V$ the pairs of approximate eigenfunctions

4.4 $$\begin{aligned} \Psi _{\alpha }^{\pm } = \tau _\alpha ^B \big ( \chi _\eta \Phi ^\pm \big ), \quad g_{\alpha }^\pm = \Pi \Psi _\alpha ^{\pm }. \end{aligned}$$

Again, the functions $\Psi _\alpha ^{\pm }$ are good approximate solutions to the eigenvalue equation for ${\mathscr {L}}_{h}^V$. Moreover, $g_\alpha ^\pm$ is exponentially close to $\Psi _\alpha ^\pm$.

### Lemma 15

If $\eta$ is small enough then

1. For all $$\alpha \in V$$,
  $$\begin{aligned} \Vert ( {\mathscr {L}}_{h}^V - \mu _\pm ) \Psi _\alpha ^\pm \Vert = {\mathscr {O}}\big ( e^{- \frac{d(L-R-3\eta )}{h}} \big ), \quad \Vert \Psi _\alpha ^\pm \Vert = 1 + {\mathscr {O}}\big ( e^{- \frac{d(L-R-3\eta )}{h}} \big ), \end{aligned}$$
  and
  $$\begin{aligned} \langle \Psi _\alpha ^+, \Psi _\alpha ^{-} \rangle = {\mathscr {O}}\big ( e^{- \frac{2d(L-R-3\eta )}{h}} \big ). \end{aligned}$$
2. For all $$\alpha \in V$$,
  $$\begin{aligned} \Vert g_\alpha ^\pm - \Psi _\alpha ^\pm \Vert = {\mathscr {O}}\big ( e^{- \frac{d(L-R-4\eta )}{h}} \big ), \quad \langle {\mathscr {L}}_{h}^V ( g_\alpha ^\pm - \Psi _\alpha ^\pm ), g_\alpha ^\pm - \Psi _\alpha ^\pm \rangle = {\mathscr {O}}\big ( e^{- \frac{d(L-R-4\eta )}{h}} \big ), \end{aligned}$$
  and the estimates are uniform with respect to $\alpha \in V$.
3. The vectors $(g_\alpha ^\pm )_{\alpha \in V}$ span a dense subspace of ${\mathscr {E}}_h$.

### Proof

The proof of the first item is identical to Lemma 10, except for the last statement. There, we simply use that $\langle \Phi ^+, \Phi ^- \rangle = 0$ and the decay of $\Phi ^{\pm }$ to find

$$\begin{aligned} \langle \Psi _\alpha ^+, \Psi _\alpha ^- \rangle = \langle (\chi _\eta ^2-1) \Phi ^+, \Phi ^- \rangle = {\mathscr {O}}\big ( e^{- \frac{2d(L-R-3\eta )}{h}} \big ). \end{aligned}$$

The second item is identical to Lemma 11. To obtain that $(g_\alpha ^\pm )_{\alpha \in V}$ span a dense subspace of ${\mathscr {E}}_h$, we also follow the same strategy. We can show that any $\Psi \in \lbrace g_\alpha ^\pm \,, \alpha \in V \rbrace ^\perp$ satisfy

$$\begin{aligned} \langle {\mathscr {L}}_{h}^V \Psi , \Psi \rangle \ge \Big ( \mu _2 + 2c_0 h^2 -C h^N\Big ) \Vert \Psi \Vert ^2, \end{aligned}$$

using the spectral projector

$$\begin{aligned} {\widetilde{\Pi }}_0 = \langle \cdot , \Phi ^+ \rangle \Phi ^+ + \langle \cdot , \Phi ^- \rangle \Phi ^- \end{aligned}$$

instead of $\Pi _0$, and the lower bound $\mu _3 - \mu _2 \ge 2 c_0 h^2$. If $\Psi \in {\mathscr {E}}_h$ we also have $\langle {\mathscr {L}}_{h}^V \Psi , \Psi \rangle \le (\mu _2 + c_0 h^2) \Vert \Psi \Vert ^2$, and therefore $\Psi$ must have vanishing norm—since $\mu _1 = \mu _2 + o(h^2)$. $\square$

The proof of Lemma 12 also works here to provide some decay on $g_\alpha ^\pm - \Psi _\alpha ^\pm$,

4.5 $$\begin{aligned} \Vert e^{ h \langle x - \alpha \rangle } ( g_\alpha ^\pm - \Psi _\alpha ^\pm ) \Vert = {\mathscr {O}}( e^{-\frac{d(L-R-4\eta )}{h}} ), \quad \Vert e^{ h \langle x - \alpha \rangle } {\mathscr {L}}_{h}^V ( g_\alpha ^\pm - \Psi _\alpha ^\pm ) \Vert = {\mathscr {O}}( e^{-\frac{d(L-R-4\eta )}{h}} ).\nonumber \\ \end{aligned}$$

We are now in position to prove the main result of this section, which is the analog of Theorem 13. We define effective operators which act on the space $\ell ^p(V) \otimes {\mathbb {C}}^2$. An element of this space will be denoted by $x= (x^+, x^-)$, where $x^+$, $x^- \in \ell ^p(V)$. Operators acting on this space can be described by an infinite matrix ${\textbf{M}} = ({\textbf{M}}_{\alpha \beta }^{\sigma \sigma '})_{\alpha \beta \in V, \sigma \sigma ' \in \lbrace \pm \rbrace }$, and $y = {\textbf{M}} x$ is then such that

$$\begin{aligned} y^\sigma _\alpha = \sum _{\beta \in V, \sigma ' \in \lbrace \pm \rbrace } {\textbf{M}}_{\alpha \beta }^{\sigma \sigma '} x^{\sigma '}_\beta . \end{aligned}$$

We recall the size of the error terms,

$$\begin{aligned} {\mathcal {E}}_{\varepsilon ,L} = e^{- \frac{2S_h(L)}{h}} + e^{-\frac{2d(L-R-4\eta )}{h}}+ e^{-\frac{S_h(L(1+\varepsilon ))}{h}} + e^{-\frac{d(L-R-4\eta ) + d(\varepsilon L + R + 2 \eta )}{h}}, \end{aligned}$$

which are controlled in Lemma 21.

### Theorem 16

Let $\varepsilon >0$ and assume $e_0=\frac{1}{2}$. For all $p \in [1,\infty ]$, the infinite matrix ${\textbf{G}} = (\langle g_\alpha ^\sigma , g_\beta ^{\sigma '} \rangle )_{\alpha \beta \in V, \sigma \sigma ' \in \lbrace \pm \rbrace }$ defines a bounded operator on $\ell ^p(V) \otimes {\mathbb {C}}^2$. Moreover,

1. We have the following estimate in $\ell ^p$ operator norm topology
  $$\begin{aligned} {\textbf{G}}= {\textbf{I}} + {\textbf{T}}+ {\mathscr {O}}({\mathcal {E}}_{\varepsilon ,L}), \quad {\text {in}}\quad {\mathcal {L}}(\ell ^p(V)\otimes {\mathbb {C}}^2), \end{aligned}$$
  where $${\textbf{T}}_{\alpha \beta }^{\sigma \sigma '} = \langle g_\alpha ^\sigma , g_\beta ^{\sigma '} \rangle {\textbf{1}}_{L \le |\alpha - \beta | \le L(1 + \varepsilon )}$$.
2. ${\textbf{G}}^{-1}$ is a bounded operator on $\ell ^p(V)$, and has a square root satisfying
  $$\begin{aligned} {\textbf{G}}^{- \frac{1}{2}} = {\textbf{I}} - \frac{1}{2} {\textbf{T}}+ {\mathscr {O}}({\mathcal {E}}_{\varepsilon ,L}), \quad {\text {in}}\quad {\mathcal {L}}(\ell ^p(V) \otimes {\mathbb {C}}^2). \end{aligned}$$
3. The vectors
  $$\begin{aligned} e_\alpha ^\sigma = \sum _{\beta \in V, \sigma ' \in \lbrace \pm \rbrace } ({\textbf{G}}^{- \frac{1}{2}})_{\alpha \beta }^{\sigma \sigma '} g_\beta ^{\sigma '}, \qquad \alpha \in V, \quad \sigma \in \lbrace \pm \rbrace , \end{aligned}$$
  define a Hilbert basis of ${\mathscr {E}}_h$.
4. The restriction of ${\mathscr {L}}_{h}^V$ to ${\mathscr {E}}_h$ is unitarily equivalent to the operator ${\textbf{M}}= ( \langle {\mathscr {L}}_{h}^V e_\alpha ^\sigma , e_\beta ^{\sigma '} \rangle )_{\alpha , \beta \in V, \sigma , \sigma ' \in \lbrace \pm \rbrace }$ acting on $\ell ^2(V) \otimes {\mathbb {C}}^2$, and
  $$\begin{aligned} {\textbf{M}}= {\textbf{D}} + {\textbf{W}} +{\mathscr {O}}({\mathcal {E}}_{\varepsilon ,L}), \end{aligned}$$
  where $${\textbf{D}}_{\alpha \beta }^{\sigma \sigma '} = \mu _\sigma {\textbf{I}}_{\alpha \beta }^{\sigma \sigma '}$$ and
  4.6 $$\begin{aligned} {\textbf{W}}_{\alpha \beta }^{\sigma \sigma '} = \big \langle ({\mathscr {L}}_{h}- \frac{\mu _\sigma + \mu _{\sigma '}}{2}) \Psi _\alpha ^\sigma , \Psi _\beta ^{\sigma '} \big \rangle {\textbf{1}}_{L \le |\alpha - \beta | \le L(1 + \varepsilon )}. \end{aligned}$$

### Proof

1. As before, we can use the $\ell ^1$ operator norm. We decompose ${\textbf{G}}$ as

$$\begin{aligned} {\textbf{G}} = \widetilde{{\textbf{I}}} + {{\textbf{T}}} + \widetilde{{\textbf{R}}}, \end{aligned}$$

where

$$\begin{aligned} \widetilde{{\textbf{I}}}_{\alpha \beta }^{\sigma \sigma '} = \langle g_\alpha ^\sigma , g_\beta ^{\sigma '} \rangle {\textbf{1}}_{\alpha = \beta }, \quad {\textbf{T}}_{\alpha \beta }^{\sigma \sigma '} = \langle g_\alpha ^\sigma , g_\beta ^{\sigma '} \rangle {\textbf{1}}_{L \le |\alpha - \beta | \le L(1+\varepsilon )}, \quad \widetilde{{\textbf{R}}}_{\alpha \beta }^{\sigma \sigma '} = \langle g_\alpha ^\sigma , g_\beta ^{\sigma '} \rangle {\textbf{1}}_{|\alpha - \beta | > L(1+\varepsilon )}. \end{aligned}$$

As in (3.22), we bound $\widetilde{{\textbf{R}}}$ and ${\textbf{I}} - \widetilde{{\textbf{I}}}$ using Lemma 15, Eq. (4.5) and Lemma 18. Note that to estimate the off-diagonal terms of $\widetilde{{\textbf{I}}}$ we need the estimate on $\langle \Psi _\alpha ^+, \Psi _\alpha ^- \rangle$ from Lemma 15.

2. The proof of the second item is identical to Theorem 13. Indeed, ${\textbf{G}}$ is still a perturbation of the identity, and a Taylor expansion of the square root gives the result.

3. The third item is also unchanged. $e_\alpha ^\sigma$ is a perturbation of $g_\alpha ^\sigma$, and these vectors span a dense subspace of ${\mathscr {E}}_h$. The family $(e_\alpha ^\sigma )_{\alpha \in V, \sigma \in \lbrace \pm \rbrace }$ is also orthonormal since

$$\begin{aligned} \langle e_\alpha ^\sigma , e_{\alpha '}^{\sigma '} \rangle&= \sum _{\beta , \beta ' \in V \tau , \tau ' \in \lbrace \pm \rbrace } ({\textbf{G}}^{- \frac{1}{2}})_{\alpha \beta }^{\sigma \tau } \langle g_\beta ^\tau , g_{\beta '}^{\tau '} \rangle ({\textbf{G}}^{- \frac{1}{2}})_{\alpha ' \beta '}^{\sigma ' \tau '} = ({\textbf{G}}^{- \frac{1}{2}} {\textbf{G}}{\textbf{G}}^{- \frac{1}{2}})_{\alpha \alpha '}^{\sigma \sigma '} = {\textbf{I}}_{\alpha \alpha '}^{\sigma \sigma '}, \end{aligned}$$

and therefore it is a Hilbert basis of ${\mathscr {E}}_h$.

4. ${\mathscr {L}}_{h}^V$ is unitarily equivalent to ${\textbf{M}}$ through the Hilbert basis $(e_\alpha ^\sigma )$. Before estimating ${\textbf{M}}$, we introduce the operator ${\widetilde{{\textbf{M}}}} = ( \langle {\mathscr {L}}_{h}^V g_\alpha ^\sigma , g_\beta ^{\sigma '} \rangle )_{\alpha \beta \in V, \sigma \sigma ' \in \lbrace \pm \rbrace }$. We claim that

4.7 $$\begin{aligned} {\widetilde{{\textbf{M}}}} = {\textbf{D}} + {\textbf{W}} + \frac{1}{2}( {\textbf{D}} {\textbf{T}} + {\textbf{T}} {\textbf{D}}) + {\mathscr {O}} \big ({\mathcal {E}}_{\varepsilon , L} \big ), \end{aligned}$$

in $\ell ^1(V) \otimes {\mathbb {C}}^2$ operator norm topology, with ${\textbf{D}}_{\alpha \beta }^{\sigma \sigma '} = \mu _\sigma {\textbf{I}}_{\alpha \beta }^{ \sigma \sigma '}$. Indeed, on the diagonal $\alpha = \beta$ we have

$$\begin{aligned} \langle {\mathscr {L}}_{h}^V g_\alpha ^\sigma , g_\alpha '^{\sigma '} \rangle&= \mu _\sigma \langle g_\alpha ^\sigma , g_\alpha ^{\sigma '} \rangle + {\mathscr {O}}(e^{-\frac{2d(L-R-4\eta )}{h}}) \\&= \mu _\sigma {\textbf{I}}_{\alpha \alpha }^{\sigma \sigma '} + {\mathscr {O}}(e^{-\frac{2d(L-R-4\eta )}{h}}) \end{aligned}$$

because of Lemma 15. For $L \le |\alpha - \beta | \le L(1+\varepsilon )$, we have

$$\begin{aligned} \langle {\mathscr {L}}_{h}^V g_\alpha ^\sigma , g_\beta ^{\sigma '} \rangle&= \langle {\mathscr {L}}_{h}\Psi _\alpha ^\sigma , \Psi _\beta ^{\sigma '} \rangle + {\mathscr {O}}(e^{-\frac{2d(L-R-4\eta )}{h}}) \\&= {\textbf{W}}_{\alpha \beta }^{\sigma \sigma '} + \frac{\mu _\sigma + \mu _{\sigma '}}{2} \langle \Psi _\alpha ^\sigma , \Psi _\beta ^{\sigma '} \rangle + {\mathscr {O}}(e^{-\frac{2d(L-R-4\eta )}{h}})\\&= {\textbf{W}}_{\alpha \beta }^{\sigma \sigma '} + \frac{\mu _\sigma + \mu _{\sigma '}}{2} {\textbf{T}}_{\alpha \beta }^{\sigma \sigma '} +{\mathscr {O}}(e^{-\frac{2d(L-R-4\eta )}{h}}), \end{aligned}$$

where we used Lemma 15 again in the first line. Finally, the remaining terms $|\alpha - \beta |> L(1+\varepsilon )$ are bounded exactly as in Theorem 13, using Eq. (4.5) and Lemma 11. This proves (4.7). Now, by definition of ${\textbf{M}}$ and $e_\alpha ^\sigma$ we have

$$\begin{aligned} {\textbf{M}}_{\alpha \alpha '}^{\sigma \sigma '} = \sum _{\beta \beta ' \in V, \tau , \tau ' \in \lbrace \pm \rbrace } ({\textbf{G}}^{- \frac{1}{2}})_{\alpha \beta }^{\sigma \tau }( {\textbf{G}}^{- \frac{1}{2}})_{\alpha ' \beta '}^{\sigma ' \tau '} {\widetilde{{\textbf{M}}}}_{\beta \beta '}^{\tau \tau '} = ({\textbf{G}}^{- \frac{1}{2}} {\widetilde{{\textbf{M}}}} {\textbf{G}}^{- \frac{1}{2}} )_{\alpha \alpha '}^{\sigma \sigma '}. \end{aligned}$$

Moreover using item (2) and (4.7) we deduce

$$\begin{aligned} {\textbf{M}}= {\textbf{G}}^{- \frac{1}{2}} {\widetilde{{\textbf{M}}}} {\textbf{G}}^{- \frac{1}{2}} = {\textbf{D}} + {\textbf{W}} + {\mathscr {O}}({\mathcal {E}}_{\varepsilon ,L}), \end{aligned}$$

in $\ell ^1$ (and thus $\ell ^2$) operator norm topology. $\square$

## Proof of Theorems 3 and 4

In this section we prove the spectral gap estimates in the case of two obstacles. The centers of the obstacles are $V= \lbrace \alpha _\ell , \alpha _r \rbrace$ with

$$\begin{aligned} \alpha _\ell = \Big ( - \frac{L}{2},0 \Big ), \qquad \alpha _r = \Big ( \frac{L}{2}, 0 \Big ). \end{aligned}$$

### Proof of Theorem 3

When $e_0 \ne \frac{1}{2}$, we use Theorem 13 with $\varepsilon \ge 2$. In this case, ${\mathscr {E}}_h$ is two-dimensional and the restriction of ${\mathscr {L}}_{h}^V$ to ${\mathscr {E}}_h$ is unitarily equivalent to the matrix

$$\begin{aligned} {\textbf{M}}= \begin{pmatrix} \mu _1 & \quad {\textbf{W}}_{\alpha _\ell \alpha _r} \\ {\textbf{W}}_{\alpha _r \alpha _\ell }& \quad \mu _1 \end{pmatrix} + {\mathscr {O}}\big ( {\mathcal {E}}_{\varepsilon ,L} \big ). \end{aligned}$$

The first two eigenvalues of ${\mathscr {L}}_{h}^V$ are thus of the form

$$\begin{aligned} \lambda _1 = \mu _1 - \big |{\textbf{W}}_{\alpha _\ell \alpha _r} \big | + {\mathscr {O}}\big ( {\mathcal {E}}_{\varepsilon ,L} \big ), \qquad \lambda _2 = \mu _1 + \big |{\textbf{W}}_{\alpha _\ell \alpha _r} \big | + {\mathscr {O}}\big ( {\mathcal {E}}_{\varepsilon ,L} \big ). \end{aligned}$$

In Lemma 19 and 20 below the interaction coefficient ${\textbf{W}}_{\alpha _\ell \alpha _r}$ is estimated and we find

$$\begin{aligned} \lambda _2 - \lambda _1&= 4 |{{\textrm{Re}}}(w_L)| + {\mathscr {O}}\big ( {\mathcal {E}}_{\varepsilon ,L} \big ) \\&= 4 |{{\textrm{Re}}}(C_L)| K_L^{-e_0-1} h e^{-\frac{S_h(L)}{h}} \big ( 1+o(1) \big )+ {\mathscr {O}}\big ( {\mathcal {E}}_{\varepsilon ,L} \big ). \end{aligned}$$

The error terms ${\mathcal {E}}_{\varepsilon ,L}$ are shown to be small enough in Lemma 21, using that $L>6R$. $\square$

### Proof of Theorem 4

When $e_0 = \frac{1}{2}$, the eigenspace ${\mathscr {E}}_h$ is four-dimensional and we use Theorem 16 instead. The Hilbert space $\ell ^2(V) \otimes {\textbf{C}}^2$ is four-dimensional as well, and ${\mathscr {L}}_{h}^V$ is unitarily equivalent to the matrix

$$\begin{aligned} {\textbf{M}}= \begin{pmatrix} \mu _-& \quad {\textbf{W}}^{--}& \quad 0 & \quad {\textbf{W}}^{-+} \\ {\textbf{W}}^{--} & \quad \mu _- & \quad \overline{{\textbf{W}}}^{+-} & \quad 0 \\ 0 & \quad {\textbf{W}}^{+-} & \quad \mu _+ & \quad {\textbf{W}}^{++} \\ \overline{{\textbf{W}}}^{-+} & \quad 0 & \quad {\textbf{W}}^{++} & \quad \mu _+ \end{pmatrix} + {\mathscr {O}}\big ( {\mathcal {E}}_{\varepsilon ,L} \big ), \end{aligned}$$

where we used the short-hand notation ${\textbf{W}}^{\sigma \sigma '} = {\textbf{W}}^{\sigma \sigma '}_{\alpha _\ell \alpha _r}$. We use the Lemmas 19 and 20 to estimate the interaction coefficients and find

$$\begin{aligned} {\textbf{M}}= \begin{pmatrix} \mu _- & \quad \lambda _h K_L^{-1} & \quad 0 & \quad -\lambda _h \\ \lambda _h K_L^{-1} & \quad \mu _- & \quad \lambda _h & \quad 0 \\ 0 & \quad \lambda _h& \quad \mu _+ & \quad - \lambda _h K_L \\ -\lambda _h & \quad 0 & \quad -\lambda _h K_L & \quad \mu _+ \end{pmatrix} + o \big ( h e^{- \frac{S_h(L)}{h}} \big ), \end{aligned}$$

with $\lambda _h =2 (-1)^{m_+} {{\textrm{Re}}}(C_L) K_L^{-e_0}$. In the special case when $\mu _- = \mu _+$ we find

$$\begin{aligned} \lambda _1&= \mu _1 - |\lambda _h| \big ( K_L + K_L^{-1} \big ) + o \big ( h e^{- \frac{S_h(L)}{h}} \big ),\\ \lambda _2&= \mu _1+ o \big ( h e^{- \frac{S_h(L)}{h}} \big ),\\ \lambda _3&= \mu _1+o \big ( h e^{- \frac{S_h(L)}{h}} \big ) ,\\ \lambda _4&= \mu _1 + |\lambda _h| \big ( K_L + K_L^{-1} \big ) +o \big ( h e^{- \frac{S_h(L)}{h}} \big ), \end{aligned}$$

and this concludes the proof of Theorem 3. $\square$

## Tunneling for a Lattice of Obstacles

In this section be calculate the tunneling in the case of infinitely many disjoint obstacles. We focus here on the special case when the obstacles are distributed along a lattice, that is $V= L {\mathbb {Z}}^2$.

### Proof of Theorem 5

Let us start with the simpler situation, when $e_0 \ne \frac{1}{2}$. We use Theorem 13, and make the specific choice $\varepsilon = 0.4$. Indeed, we want $\varepsilon < \sqrt{2}-1$ to ensure that we do not capture the interaction between obstacles along diagonals. For instance, the lattice points (0, 0) and (*L*, *L*) have distance $L\sqrt{2} > L(1+ \varepsilon )$ to each other: their interaction is not included in the infinite matrix ${\textbf{W}}$. We also want $\varepsilon$ as large as possible in order to reduce the size of the error terms ${\mathcal {E}}_{\varepsilon ,L}$. Note that we could also choose $\varepsilon$ larger and take into account the diagonal interactions, but this would make the interaction matrix ${\textbf{W}}$ more complicated.

With the choice $\varepsilon = 0.4$, we obtain that the restriction of ${\mathscr {L}}_{h}^V$ to ${\mathscr {E}}_h$ is unitarily equivalent to an operator ${\textbf{M}}$ on $\ell ^2(L{\mathbb {Z}}^2)$ of the form

$$\begin{aligned} {\textbf{M}}= \mu _1 {\textbf{I}} + {\textbf{W}} + {\mathscr {O}}({\mathcal {E}}_{\varepsilon ,L}). \end{aligned}$$

For $\alpha$, $\beta \in L {\mathbb {Z}}^2$, the coefficients ${\textbf{W}}_{\alpha \beta }$ are all vanishing unless $|\alpha - \beta |=L$ (i.e. they are closest neighbours). In this case, by Lemmas 19 and 20 we have

$$\begin{aligned} {\textbf{W}}_{\alpha \beta } = (-1)^{m_-+1} e^{\frac{iB}{2h}\alpha \wedge \beta } 2C_L K_L^{-e_0-1} h e^{- \frac{S_h(L)}{h}} \big ( 1+ o(1) \big ). \end{aligned}$$

With the notations of Theorem 5 this gives

$$\begin{aligned} {\textbf{W}} = \lambda {\textbf{X}}+ o \big ( h e^{-\frac{S_h(L)}{h}} \big ), \end{aligned}$$

where

$$\begin{aligned} {\textbf{X}}_{\alpha \beta } = {\left\{ \begin{array}{ll} e^{\frac{iB}{2h}\alpha \wedge \beta } & \quad \mathrm{{if}} \, |\alpha - \beta |=L,\\ 0 & \quad \mathrm{{otherwise}}. \end{array}\right. } \end{aligned}$$

The errors ${\mathcal {E}}_{\varepsilon ,L}$ are shown to be small enough in Lemma 21, as soon as $L > 25 R$.

We now consider $e_0 = \frac{1}{2}$. In this case we use Theorem 16 (still with $\varepsilon = 0.4$) and we obtain an operator on $\ell ^2(L{\mathbb {Z}}^2) \otimes {\textbf{C}}^2$ with similar expansion,

$$\begin{aligned} {\textbf{M}}= {\textbf{D}} + {\textbf{W}} + {\mathscr {O}}({\mathcal {E}}_{\varepsilon ,L}). \end{aligned}$$

The coefficients ${\textbf{W}}_{\alpha \beta }^{\sigma \sigma '}$ are vanishing unless $|\alpha - \beta |=L$, in which case we have

$$\begin{aligned} {\textbf{W}}_{\alpha \beta }^{\sigma \sigma '} = (-1)^{m_{\sigma '}+1} e^{\frac{iB}{2h}\alpha \wedge \beta } e^{- i \frac{\sigma - \sigma '}{2} \arg (\beta -\alpha )} 2C_L K_L^{\frac{\sigma + \sigma ' -1}{2}} h e^{-\frac{S_h(L)}{h}} \big ( 1+o(1) \big ), \end{aligned}$$

by Lemmas 19 and 20. With the notations of Theorem 5 we have ${\textbf{W}} = \lambda {\textbf{Y}}+ o \big ( h e^{-\frac{S_h(L)}{h}} \big )$. Finally, the errors ${\mathcal {E}}_{\varepsilon ,L}$ are small enough by Lemma 21. $\square$

## Data Availability

Data sharing not applicable to this article as no datasets were generated or analysed during the current study.

## Appendix A. Analysis of the Phase

The phase function involved in several integrals in this Appendix is

A.1 $$\begin{aligned} \begin{aligned} s_h^{\sigma \sigma '}(x_1,x_2)&= \frac{B}{4} \Big (x_1+\frac{\ell }{2} \Big )^2 + \frac{B}{4}\Big (x_1- \frac{\ell }{2}\Big )^2 + \frac{B}{2} x_2^2 - \frac{BR^2}{2} + i \frac{B\ell x_2}{2} \\  &\qquad - hm_{\sigma } \log \Big ( \frac{\ell /2 +x_1+ix_2}{R} \Big ) - hm_{\sigma '} \log \Big (\frac{ \ell /2 - x_1 +ix_2}{R} \Big ), \end{aligned} \end{aligned}$$

where $\sigma$, $\sigma ' \in \lbrace \pm \rbrace$. It depends on the parameter $\ell > 2R$. Recalling that $m_\pm$ depends on *h*, we consider the main part of this function,

A.2 $$\begin{aligned} \begin{aligned} s_0(x_1,x_2)&= \frac{B}{4} \Big (x_1+\frac{\ell }{2} \Big )^2 + \frac{B}{4}\Big (x_1- \frac{\ell }{2}\Big )^2 + \frac{B}{2} x_2^2 - \frac{BR^2}{2} + i \frac{B\ell x_2}{2} \\  &\qquad -\frac{BR^2}{2} \log \Big ( \frac{\ell /2 +x_1+ix_2}{R} \Big ) - \frac{BR^2}{2} \log \Big (\frac{ \ell /2 - x_1 +ix_2}{R} \Big ). \end{aligned} \end{aligned}$$

We collect below the main properties of these functions, necessary to use the Laplace method.

### Lemma 17

The function $(x_1,x_2) \mapsto s_0(x_1,x_2)$ has the following properties.

1. $s_0$ is holomorphic on $D = \lbrace {{\textrm{Re}}}(x_1) \in \big ( - \frac{\ell }{2}, \frac{\ell }{2} \big )$, ${{\textrm{Im}}}(x_2) \le 0 \rbrace \subset {\textbf{C}}^2$.
2. $s_0$ has a unique critical point $(x_1^*,x_2^*)$ in *D*, which is
  $$\begin{aligned} x_1^* = 0, \quad x_2^* = -i \sqrt{\frac{\ell ^2}{4}-R^2}. \end{aligned}$$
3. The critical value is
  $$\begin{aligned} s_0(x_1^*,x_2^*) = \frac{B \ell }{4}\sqrt{\ell ^2-4R^2} - BR^2 \ln \Big ( \frac{\ell + \sqrt{\ell ^2-4R^2}}{2R} \Big ). \end{aligned}$$
4. The Hessian is such that ${{\textrm{Im}}} \nabla ^2 s_0(x^*) = 0$. Also, $\partial _1 \partial _2 s_0(x^*) = 0$ and
  $$\begin{aligned} {\left\{ \begin{array}{ll} \partial _1^2 s_0(x^*) = \frac{B\ell }{2R^2}\big ( \ell - \sqrt{\ell ^2-4R^2} \big ), \\ \partial _2^2 s_0(x^*) = \frac{B}{2R^2}\sqrt{\ell ^2-4R^2} \big ( \ell - \sqrt{\ell ^2-4R^2} \big ). \end{array}\right. } \end{aligned}$$
5. The function $x_1 \in (-\frac{\ell }{2}, \frac{\ell }{2}) \mapsto s_0(x_1,x_2^*)$ is real valued, and has a unique global minimum at 0.
6. For $x_2 \in {\textbf{R}}$, we have the following expansion as $x_2 \rightarrow 0$,
  $$\begin{aligned} s_h^{\sigma \sigma '}(0,x_2+x_2^*)&= s_h^{\sigma \sigma '}(0,x_2^*) + \frac{B \sqrt{\ell ^2-4R^2}}{4R^2} ( \ell - \sqrt{\ell ^2-4R^2} ) x_2^2 \\  &\qquad - i \frac{x_2}{R} (\ell - \sqrt{\ell ^2-4R^2}) \sqrt{h \Theta _0 B} + {\mathscr {O}}(x_2^3 + \sqrt{h} x_2^2 + h x_2), \end{aligned}$$
  and
  $$\begin{aligned} s_h^{\sigma \sigma '}(0,x_2^*)&= \frac{B\ell }{4} \sqrt{\ell ^2-4R^2} - h (m_\sigma + m_{\sigma '}) \ln \Big ( \frac{ \ell + \sqrt{\ell ^2-4R^2} }{2R} \Big ). \\&= S_h(\ell ) - h ( 2 {\mathcal {C}}_2 + m_\sigma + m_{\sigma '} - 2 \xi _h) \ln \Big ( \frac{ \ell + \sqrt{\ell ^2-4R^2} }{2R} \Big ), \end{aligned}$$
  where we recall
  A.3 $$\begin{aligned} S_h(\ell ) = \frac{B\ell }{4}\sqrt{\ell ^2 - 4R^2} - \big ( BR^2 + 2\sqrt{h\Theta _0 BR^2} \big ) \ln \Big ( \frac{\ell + \sqrt{\ell ^2 - 4R^2}}{2R}\Big ). \end{aligned}$$

## Appendix B. Estimates on the Interaction Coefficients

We collect in this appendix estimates on the scalar products

$$\begin{aligned} \langle \Psi _\alpha , \Psi _\beta \rangle , \qquad \langle \Psi _\alpha ^\sigma , \Psi _\beta ^{\sigma '} \rangle , \end{aligned}$$

and on the interaction coefficients

$$\begin{aligned} {\textbf{W}}_{\alpha \beta } = \langle \big ( {\mathscr {L}}_{h}- \mu _1 \big ) \Psi _\alpha , \Psi _\beta \rangle , \qquad {\textbf{W}}_{\alpha \beta }^{\sigma \sigma '} = \big \langle \big ( {\mathscr {L}}_{h}- \frac{\mu _\sigma + \mu _{\sigma '}}{2} \big ) \Psi _\alpha ^\sigma , \Psi _\beta ^{\sigma '} \big \rangle , \end{aligned}$$

where $\alpha$, $\beta \in V$ and $\sigma$, $\sigma ' \in \lbrace \pm \rbrace$. These estimates follow from the Laplace method, and the involved phase $s_h^{\sigma \sigma '}$ was described in Appendix A. We start by estimating the scalar products in Lemma 18. We then explain how to rewrite the interaction coefficients by an interaction by part argument in Lemma 19. We finally estimate these coefficients in Lemma 20.

### Lemma 18

Let $\alpha$, $\beta \in V$ such that $|\alpha - \beta | = \ell \ge L$. Then our quasimodes $\Psi _\alpha$ and $\Psi _\alpha ^\pm$ are such that:

1. When $e_0 \ne \frac{1}{2}$ we have
  $$\begin{aligned} \langle \Psi _\alpha , \Psi _{\beta } \rangle = {\mathscr {O}} \big (h^{\frac{1}{2}} e^{-\frac{S_h(\ell )}{h}} + e^{-\frac{d(L-R-3\eta ) + d(\ell - L + R + 2 \eta )}{h}} \big ), \end{aligned}$$
  as $h \rightarrow 0$ along a $e_0$-sequence, and uniformly with respect to $\alpha$ and $\beta$.
2. When $e_0 = \frac{1}{2}$, for $\sigma$, $\sigma ' \in \lbrace \pm \rbrace$ we have
  $$\begin{aligned} \langle \Psi _\alpha ^\sigma , \Psi _{\beta }^{\sigma '} \rangle = {\mathscr {O}} \big (h^{\frac{1}{2}} e^{-\frac{S_h(\ell )}{h}} + e^{-\frac{d(L-R-3\eta ) + d(\ell - L + R + 2 \eta )}{h}} \big ), \end{aligned}$$
  as $h \rightarrow 0$ along a $\frac{1}{2}$-sequence, and uniformly with respect to $\alpha$ and $\beta$.

### Proof

Note that when $e_0 \ne \frac{1}{2}$, $\Psi _\alpha$ is equal to $\Psi _\alpha ^{-}$. Therefore, we can focus on the second case. We first use the definition (4.4) of $\Psi _\alpha ^\sigma$ and properties of the magnetic translations, in order to center the integral at 0. Indeed,

$$\begin{aligned} \langle \Psi _\alpha ^\sigma , \Psi _\beta ^{\sigma '} \rangle&= \langle \tau _\alpha ^B \chi _\eta \Phi ^\sigma , \tau _\beta ^B \chi _\eta \Phi ^{\sigma '}\rangle = \langle \tau ^B_{-\frac{\alpha + \beta }{2}} \tau ^B_\alpha \chi _\eta \Phi ^\sigma , \tau ^B_{-\frac{\alpha + \beta }{2}} \tau ^B_\beta \chi _\eta \Phi ^{\sigma '} \rangle \\&=e^{\frac{iB}{2h} \alpha \wedge \beta } \langle \tau _{\frac{\alpha - \beta }{2}}^B \chi _\eta \Phi ^\sigma , \tau ^B_{\frac{\beta - \alpha }{2}} \chi _\eta \Phi ^{\sigma '} \rangle . \end{aligned}$$

Now we use a rotation $R_{-\theta }$ such that $R_{-\theta } \big ( \frac{\alpha - \beta }{2} \big ) = (- \frac{\ell }{2}, 0)$. The commutation with magnetic translations gives

$$\begin{aligned} R_\theta \cdot \tau _{\alpha }^B = \tau _{(R_{-\theta } \alpha )}^B \cdot R_\theta , \end{aligned}$$

and therefore

$$\begin{aligned} \langle \Psi _\alpha ^\sigma , \Psi _\beta ^{\sigma '} \rangle = e^{\frac{iB}{2h} \alpha \wedge \beta } \langle \tau ^B_{(- \frac{\ell }{2},0)} R_\theta \chi _\eta \Phi ^\sigma , \tau ^B_{( \frac{\ell }{2},0)} R_\theta \chi _\eta \Phi ^{\sigma '}\rangle . \end{aligned}$$

Then, using the angular dependence of $\Phi ^\sigma$ we have $R_\theta \Phi ^\sigma (x) =\Phi ^\sigma ( R_\theta x) = e^{im_\sigma \theta } \Phi ^\sigma (x)$ and

B.1 $$\begin{aligned} \langle \Psi _\alpha ^\sigma , \Psi _\beta ^{\sigma '} \rangle = e^{\frac{iB}{2h} \alpha \wedge \beta + i (m_\sigma - m_{\sigma '})\theta } \langle \tau ^B_{(-\frac{\ell }{2},0)} \chi _\eta \Phi ^\sigma , \tau ^B_{(\frac{\ell }{2},0)} \chi _\eta \Phi ^{\sigma '} \rangle . \end{aligned}$$

We are reduced to the case of two disks on the horizontal axis, with relative distance $\ell$. For simplicity we write $\epsilon = e^{i(m_\sigma - m_{\sigma '})\theta }$, which is either equal to 1 or $e^{\pm i\theta }$. Now we can use the explicit formula for $\Phi ^\sigma$ from Lemma 7,

B.2 $$\begin{aligned} \Phi ^\sigma (x) = \Big ( \frac{BR^2}{h} \Big )^{\frac{\Theta _0}{4}} \Big ( \frac{z}{R} \Big )^{m_\sigma } e^{- \frac{B}{4h}(|z|^2 - R^2)} u_h(|z|), \end{aligned}$$

where we identify $x=(x_1,x_2)$ and $z=x_1+ix_2$. Replacing we find

$$\begin{aligned}  &   \langle \Psi _\alpha ^\sigma , \Psi _\beta ^{\sigma '} \rangle = e^{\frac{iB}{2h} \alpha \wedge \beta } e^{i \pi m_{\sigma '}} \epsilon \Big ( \frac{BR^2}{h} \Big )^{\frac{\Theta _0}{2}} \int _{{\mathbb {R}}^2} e^{- \frac{iB\ell x_2}{2h}} \chi _\eta \Big (z+ \frac{\ell }{2} \Big ) \chi _\eta \Big (z-\frac{\ell }{2} \Big ) \\  &   \quad \Big (\frac{\ell /2 +z}{R}\Big )^{m_\sigma } \overline{\Big (\frac{ \ell /2 - z}{R} \Big )^{m_{\sigma '}}} e^{- \frac{B}{4h} ( |z+\frac{\ell }{2}|^2 + |z- \frac{\ell }{2}|^2-2R^2)} u_h\Big (\big |z+\frac{\ell }{2}\big |\Big )u_h \Big (\big |z-\frac{\ell }{2}\big |\Big ) \textrm{d}z. \end{aligned}$$

For simplicity we will not specify the arguments of $\chi _\eta$ in the notation below. Also, we can write the power of any complex number as exponential of a logarithm. The formula holds for any choice of complex logarithm, but on the support of $\chi _\eta$, the standard logarithm is continuous—and thus, we make this choice. We obtain

$$\begin{aligned} | \langle \Psi _\alpha ^{\sigma }, \Psi _\beta ^{\sigma '} \rangle | =\Big ( \frac{BR^2}{h} \Big )^{\frac{\Theta _0}{2}} \Big | \int _{{\mathbb {R}}^2} e^{- \frac{s_h^{\sigma \sigma '}(z)}{h}} \chi _\eta u_h \Big (\big |z+\frac{\ell }{2}\big |\Big ) \chi _\eta u_h \Big (\big |z-\frac{\ell }{2}\big |\Big ) \textrm{d}z \Big |, \end{aligned}$$

with phase

$$\begin{aligned} s_h^{\sigma \sigma '}(x_1,x_2)&= \frac{B}{4} \big |z+\frac{\ell }{2} \big |^2 + \frac{B}{4}\big |z- \frac{\ell }{2}\big |^2 - \frac{BR^2}{2} + i \frac{B\ell x_2}{2} \\  &\qquad - hm_\sigma \log \Big ( \frac{\ell /2 +z}{R} \Big ) - hm_{\sigma '} \log \Big (\frac{ \ell /2 - {\bar{z}}}{R} \Big ) . \end{aligned}$$

Note that ${{\textrm{Re}}}(s_h^{\sigma \sigma '}(z)) \ge d \Big (\big |z+\frac{\ell }{2} \big | \Big ) + d \Big (\big |z-\frac{\ell }{2} \big | \Big )$. We want to remove the cutoff function $\chi _\eta$. To do so, we use that on the support of $1- \chi _\eta$ we have

$$\begin{aligned}{{\textrm{Re}}}(s_h^{\sigma \sigma '}(z)) \ge d(L-R-2\eta ) + d(\ell - L + R + 2 \eta ).\end{aligned}$$

For the same reason, we can add a cutoff ${\tilde{\chi }}(x_1)$ supported on $|x_1|< \frac{\ell }{2} - R - \eta$ and we get

$$\begin{aligned} | \langle \Psi _\alpha ^\sigma , \Psi _\beta ^{\sigma '} \rangle |&= \Big ( \frac{BR^2}{h} \Big )^{\frac{\Theta _0}{2}} \Big | \int _{{\mathbb {R}}^2} {\tilde{\chi }}(x_1) e^{- \frac{s_h^{\sigma \sigma '}(z)}{h}} u_h \Big (\big |z+\frac{\ell }{2}\big |\Big ) u_h \Big (\big |z-\frac{\ell }{2}\big |\Big ) \textrm{d}z \Big | \\  &\qquad + {\mathscr {O}}(e^{-\frac{d(L-R-3\eta ) + d(\ell - L + R + 2 \eta )}{h}}). \end{aligned}$$

This last cutoff is added to make sure the complex logarithms remain continuous. We can estimate this integral using the complex Laplace method. The phase $s_h^{\sigma \sigma '}(x_1,x_2)$ is analyzed in Lemma 17. It has a unique critical point of the form $(0,x_2^*)$ where $x_2^*$ is purely imaginary. The integral being analytic as function of $x_2$, we can make the complex shift $x_2 \mapsto x_2 + x_2^*$,

$$\begin{aligned} | \langle \Psi _\alpha ^\sigma , \Psi _\beta ^{\sigma '} \rangle |&=\Big ( \frac{BR^2}{h} \Big )^{\frac{\Theta _0}{2}} \Big | \int _{{\mathbb {R}}^2} {\tilde{\chi }}(x_1) e^{- \frac{s_h^{\sigma \sigma '}(x_1,x_2+x_2^*)}{h}} u_h \Big (M_{-}(x) \Big ) u_h \Big (M_{+}(x)\Big ) \textrm{d}x \Big |\\  &\qquad + {\mathscr {O}}(e^{-\frac{d(L-R-3\eta )+ d(\ell - L + R + 2 \eta )}{h}}), \end{aligned}$$

where $M_{\pm }(x) =\sqrt{(x_1 \pm \frac{\ell }{2} )^2 + (x_2 + x_2^*)^2}$. By Lemma 17 we have

$$\begin{aligned} s_h^{\sigma \sigma '}(x+x^*) = S_h(\ell ) + \frac{1}{2} \nabla ^2 s_0(x^*) (x,x) + iC \sqrt{h} x_2 + {\mathscr {O}}(h|x| + |x|^3), \end{aligned}$$

and using the Laplace method,

$$\begin{aligned} | \langle \Psi _\alpha ^\sigma , \Psi _\beta ^{\sigma '} \rangle | = {\mathscr {O}} \Big ( h^{\frac{1}{2}} e^{-\frac{S_h(\ell )}{h}} \Big ) + {\mathscr {O}}\Big (e^{-\frac{d(L-R-3\eta )+ d(\ell - L + R + 2 \eta )}{h}}\Big ), \end{aligned}$$

where we used that $M_{\pm }(x) = R \pm \frac{\ell x_1}{2R} + \frac{x_2^* x_2}{R} + {\mathscr {O}}(x^2)$ and (2.8). $\square$

### Lemma 19

Let $\alpha$, $\beta \in V$ be such that $L \le |\alpha - \beta | = \ell \le L(1+\varepsilon )$. Then:

1. When $e_0 \ne \frac{1}{2}$ we have
  $$\begin{aligned} {\textbf{W}}_{\alpha \beta } = (-1)^{m_-} e^{\frac{iB}{2h} \alpha \wedge \beta } 2w_\ell ^{--} + {\mathscr {O}}({\mathcal {E}}') \end{aligned}$$
  where
  $$\begin{aligned} w_\ell ^{--} = h^2 \int _{{\mathbb {R}}} e^{-\frac{iB\ell x_2}{2h}} \Phi ^- \Big (\frac{\ell }{2}, x_2 \Big ) \partial _1 \Phi ^- \Big ( \frac{\ell }{2}, x_2\Big ) \textrm{d}x_2, \end{aligned}$$
  and $${\mathcal {E}}' = h^{\frac{5}{2}} e^{- \frac{S_h(\ell )}{h}} + e^{-\frac{d(L-R-3\eta ) + d(\ell - L + R + 2 \eta )}{h}} + e^{-\frac{2d(L-R-3\eta )}{h}}$$.
2. When $e_0 = \frac{1}{2}$ we have
  $$\begin{aligned} {\textbf{W}}_{\alpha \beta }^{\sigma \sigma '} =(-1)^{m_{\sigma '}} e^{\frac{iB}{2h} \alpha \wedge \beta } e^{i (m_\sigma - m_{\sigma '}) \textrm{arg}(\beta - \alpha )} \big ( w^{\sigma \sigma '}_\ell + w^{\sigma ' \sigma }_\ell \big ) + {\mathscr {O}}({\mathcal {E}}'), \end{aligned}$$
  where
  $$\begin{aligned} w^{\sigma \sigma '}_\ell = h^2 \int _{{\mathbb {R}}} e^{-\frac{iB\ell x_2}{2h}} \Phi ^\sigma \Big (\frac{\ell }{2}, x_2 \Big ) \partial _1 \Phi ^{\sigma '} \Big ( \frac{\ell }{2}, x_2\Big ) \textrm{d}x_2. \end{aligned}$$

### Proof

When $e_0 \ne \frac{1}{2}$ the coefficient ${\textbf{W}}_{\alpha \beta }$ is equal to ${\textbf{W}}_{\alpha \beta }^{--}$. Thus, we can focus on item (2). We use translations and rotations to center the problem as in the proof of Lemma 18. This amounts to change $\alpha$ to $(- \frac{\ell }{2},0)$ and $\beta$ to $(\frac{\ell }{2},0)$. We obtain, with the notation ${\bar{\mu }} = \frac{\mu _\sigma + \mu _{\sigma '}}{2}$,

$$\begin{aligned} \langle ({\mathscr {L}}_{h}- {{\bar{\mu }}}) \Psi _\alpha ^\sigma , \Psi _\beta ^{\sigma '} \rangle&= \langle ({\mathscr {L}}_{h}- {{\bar{\mu }}}) \tau _\alpha ^B \chi _\eta \Phi ^\sigma , \tau _\beta ^B \chi _\eta \Phi ^{\sigma '} \rangle \\&=e^{\frac{iB}{2h} \alpha \wedge \beta }\langle ({\mathscr {L}}_{h}- {{\bar{\mu }}}) \tau _{\frac{\alpha - \beta }{2}}^B \chi _\eta \Phi ^\sigma , \tau _{\frac{\beta - \alpha }{2}}^B \chi _\eta \Phi ^{\sigma '} \rangle \\&=e^{\frac{iB}{2h} \alpha \wedge \beta + i (m_\sigma - m_{\sigma '})arg(\beta - \alpha )} \langle ({\mathscr {L}}_{h}- {{\bar{\mu }}}) \tau _{(-\frac{\ell }{2},0)}^B \chi _\eta \Phi ^\sigma , \tau _{(\frac{\ell }{2},0)}^B \chi _\eta \Phi ^{\sigma '} \rangle . \end{aligned}$$

We now introduce the notation $\chi _\ell (x) = \chi _\eta (x_1 + \frac{\ell }{2}, x_2)$, $\chi _r(x) = \chi _\eta (x_1 - \frac{\ell }{2},x_2)$, and $\Phi _\ell ^\sigma = \tau _{(-\frac{\ell }{2},0)}^B \Phi ^\sigma$, $\Phi _r^{\sigma '} = \tau _{(\frac{\ell }{2},0)}^B \Phi ^{\sigma '}$, so that

B.3 $$\begin{aligned} \langle ({\mathscr {L}}_{h}- {{\bar{\mu }}}) \Psi _\alpha ^\sigma , \Psi _\beta ^{\sigma '} \rangle = e^{\frac{iB}{2h}\alpha \wedge \beta } e^{i(m_\sigma - m_{\sigma '})arg(\beta - \alpha )} {\tilde{w}}^{\sigma \sigma '}, \end{aligned}$$

with ${\tilde{w}}^{\sigma \sigma '} = \langle ({\mathscr {L}}_{h}- {{\bar{\mu }}}) \chi _\ell \Phi _\ell ^{\sigma }, \chi _r \Phi _r^{\sigma '} \rangle$. We can rewrite ${\tilde{w}} ^{\sigma \sigma '}$ using the integration by part argument from [15]. Since $\Phi _\ell ^\sigma$ is an eigenfunction for the single-obstacle problem, we have

$$\begin{aligned} {\tilde{w}}^{\sigma \sigma '}&=\langle \big [ {\mathscr {L}}_{h}, \chi _\ell ] \Phi _\ell ^\sigma , \chi _r \Phi _r^{\sigma '} \rangle + \big ( \mu _\sigma - {{\bar{\mu }}} \big ) \langle \chi _\ell \Phi ^\sigma _\ell , \chi _r \Phi ^{\sigma '}_{r} \rangle \\&= \langle \big ( p_h \cdot \big [ p_h, \chi _\ell \big ] + [p_h, \chi _\ell \big ] p_h \big ) \Phi _\ell ^{\sigma } , \chi _r \Phi _r^{\sigma '} \rangle + \frac{\mu _\sigma - \mu _{\sigma '}}{2} \langle \chi _\ell \Phi ^\sigma _\ell , \chi _r \Phi ^{\sigma '}_{r} \rangle . \end{aligned}$$

The second term is estimated using $|\mu _\sigma - \mu _{\sigma '}| \le Ch^2$ and Lemma 18. Hence,

B.4 $$\begin{aligned} {\tilde{w}}^{\sigma \sigma '} = \langle \big ( p_h \cdot \big [ p_h, \chi _\ell \big ] + [p_h, \chi _\ell \big ] p_h \big ) \Phi _\ell ^{\sigma }, \chi _r \Phi _r^{\sigma '} \rangle + {\mathcal {E}}, \end{aligned}$$

with ${\mathcal {E}} = {\mathscr {O}}( h^{5/2} e^{-\frac{S_h(\ell )}{h}} + e^{-\frac{d(L-R-3\eta ) + d(\ell - L + R + 2 \eta )}{h}})$. A partial integration gives

$$\begin{aligned} {\tilde{w}}^{\sigma \sigma '}&= \langle \big [p_h, \chi _\ell \big ] \Phi _\ell ^{\sigma }, p_h \chi _r \Phi _r^{\sigma '} \rangle + \langle \big [ p_h, \chi _\ell \big ] p_h \Phi _\ell ^{\sigma }, \chi _r \Phi _r^{\sigma '} \rangle + {\mathcal {E}} \\&= -i h \int _{{\mathbb {R}}^2} (\nabla \chi _\ell ) \Phi _\ell ^{\sigma } \cdot \overline{p_h(\chi _r \Phi _r^{\sigma '})} + p_h \Phi _\ell ^{\sigma } \cdot \overline{(\nabla \chi _\ell ) \chi _r \Phi _r^{\sigma '}} \textrm{d}x + {\mathcal {E}}. \end{aligned}$$

We want replace $\chi _r$ by 1 and add a ${\textbf{1}}_{x_1 >0 }$ using the support of $\nabla \chi _\ell$. This is not free, but on the support of $\nabla \chi _{\ell }$, if either $x_1<0$ or $\chi _r \ne 1$, then $\Phi _\ell ^{\sigma }$ and $\Phi _r^{\sigma '}$ are both smaller than $e^{-\frac{d(L-R-2\eta )}{h}}$ (Lemma 7). Therefore,

$$\begin{aligned} {\tilde{w}}^{\sigma \sigma '} = -i h \int _{x_1>0} (\nabla \chi _\ell ) \Phi _\ell ^{\sigma } \cdot \overline{P \Phi _r^{\sigma '}} + P \Phi _\ell ^{\sigma } \cdot \overline{(\nabla \chi _\ell ) \Phi _r^{\sigma '}} \textrm{d}x + {\mathcal {E}} + {\mathscr {O}}(e^{-\frac{2d(L-R-3\eta )}{h}}). \end{aligned}$$

We integrate by part again, and obtain a boundary term

$$\begin{aligned} {\tilde{w}}^{\sigma \sigma '}&= i h \int _{x_1>0} \chi _\ell \nabla ( \Phi _\ell ^{\sigma } \overline{P \Phi _r^{\sigma '}}) \textrm{d}x + i h \int _{x_1=0} \chi _\ell \Phi _\ell ^{\sigma } \overline{(-ih \partial _1 + B x_2 /2) \Phi _r^{\sigma '}} \textrm{d}x_2 \\  &\quad +i h \int _{x_1>0} \chi _\ell \nabla ( \overline{\Phi _r^{\sigma '}} P \Phi _\ell ^\sigma ) \textrm{d}x +i h \int _{x_1=0} \chi _\ell (-ih\partial _1+ B x_2 /2) \Phi _\ell ^\sigma \overline{\Phi _r^{\sigma '}} \textrm{d}x_2 \\  &\quad + {\mathcal {E}} + {\mathscr {O}}(e^{-\frac{2d(L-R-3\eta )}{h}}). \end{aligned}$$

Note that

$$\begin{aligned} -ih \Big ( \nabla ( \Phi _\ell ^\sigma \overline{P\Phi _r^{\sigma '}} ) + \nabla ( \overline{\Phi _r^{\sigma '}} P\Phi _\ell ^\sigma ) \Big ) = \overline{\Phi _r^{\sigma '}} {\mathscr {L}}_{h}\Phi _\ell ^\sigma - \Phi _\ell ^\sigma \overline{{\mathscr {L}}_{h}\Phi _r^{\sigma '}} = (\mu _\sigma - \mu _{\sigma '}) \Phi _\ell ^\sigma \overline{\Phi _r^{\sigma '}}, \end{aligned}$$

because $\Phi _\ell ^\sigma$ and $\Phi _r^{\sigma '}$ are eigenfunctions of ${\mathscr {L}}_{h}^0$. We deduce

$$\begin{aligned} {\tilde{w}}^{\sigma \sigma '}&= h^2 \int _{x_1=0} \chi _\ell \big (\overline{\Phi _r^{\sigma '}} \partial _1 \Phi _\ell ^\sigma - \Phi _\ell ^\sigma \overline{\partial _1 \Phi _r^{\sigma '}} \big ) \textrm{d}x_2 + (\mu _\sigma - \mu _{\sigma '}) \int _{x_1 >0} \Phi _\ell ^{\sigma }\overline{ \Phi _r^{\sigma '}} \textrm{d}x \\  &\qquad + {\mathcal {E}} + {\mathscr {O}}( e^{-\frac{2d(L-R-3\eta )}{h}}). \end{aligned}$$

The second integral can be estimated as ${\mathcal {E}}$ using $|\mu _\sigma - \mu _{\sigma '}| \le C h^2$ and Lemma 18 again. In the first integral, we have $\chi _\ell (0,x_2) = 1$ unless both $\Phi _\ell ^{\sigma }(0,x_2)$ and $\Phi _r^{\sigma '}(0,x_2)$ are smaller than $e^{-\frac{d(L-R-2\eta )}{h}}$ and therefore

$$\begin{aligned} {\tilde{w}}^{\sigma \sigma '}&= h^2 \int _{{\mathbb {R}}} e^{-\frac{iB \ell x_2}{2h}} \Big ( \overline{\Phi ^{\sigma '}}\Big (-\frac{\ell }{2}, x_2 \Big ) \partial _1 \Phi ^\sigma \Big ( \frac{\ell }{2}, x_2 \Big ) -\Phi ^\sigma \Big (\frac{\ell }{2}, x_2 \Big ) \overline{\partial _1 \Phi ^{\sigma '}}\Big ( - \frac{\ell }{2}, x_2 \Big ) \Big ) \textrm{d}x_2 \\&\quad + {\mathscr {O}}({\mathcal {E}} + e^{-\frac{2d(L-R-3\eta )}{h}}). \end{aligned}$$

We use that $\overline{\Phi ^\sigma }(x_1,x_2) = \Phi ^\sigma (x_1,-x_2) = (-1)^{m_\sigma } \Phi ^\sigma (-x_1,x_2)$ to obtain

$$\begin{aligned} {\tilde{w}}^{\sigma \sigma '} = (-1)^{m_{\sigma '}} \big ( w^{\sigma \sigma '}_\ell + w^{\sigma ' \sigma }_\ell \big ) + {\mathscr {O}}({\mathcal {E}} + e^{-\frac{2d(L-R-3\eta )}{h}}). \end{aligned}$$

The result follows using (B.3) and (4.6). $\square$

### Lemma 20

For all $\ell \ge L$, let $K_\ell = \frac{\ell }{2R} + \sqrt{\frac{\ell ^2}{4 R^2} -1}$. There exists a $C_\ell \in {\textbf{C}}$ independent of *h* and with non-vanishing real part such that:

1. When $$e_0 \in \big ( - \frac{1}{2}, \frac{1}{2} \big ]$$,
  $$\begin{aligned} w_\ell ^{--} = C_\ell K_\ell ^{-2e_0} h e^{-\frac{S_h(\ell )}{h}} (1+o(1)), \end{aligned}$$
  as $h \rightarrow 0$ along a $e_0$-sequence.
2. When $$e_0 \in \big ( - \frac{1}{2}, \frac{1}{2} \big ]$$,
  $$\begin{aligned} w_\ell ^{++} = C_\ell K_\ell ^{2e_0} h e^{-\frac{S_h(\ell )}{h}} (1+o(1)), \end{aligned}$$
  as $h \rightarrow 0$ along a $e_0$-sequence.
3. When $$e_0 = \frac{1}{2}$$ and $$\sigma \ne \sigma '$$,
  $$\begin{aligned} w_\ell ^{\sigma \sigma '} = C_\ell h e^{-\frac{S_h(\ell )}{h}} (1+o(1)), \end{aligned}$$
  as $h \rightarrow 0$ along a $\frac{1}{2}$-sequence.

### Proof

We use the explicit formula from Lemma 7,

B.5 $$\begin{aligned} \Phi ^\sigma (x_1,x_2) = \Big ( \frac{BR^2}{h} \Big )^{ \frac{\Theta _0}{4}} \exp \Big ( - \frac{\varphi _\sigma (x)}{h} \Big ) u_h(|x|), \end{aligned}$$

with $$\varphi _\sigma (x) = \frac{B}{4}(x_1^2 + x_2^2 - R^2) - h m_\sigma \ln \big ( \frac{x_1+ix_2}{R} \big )$$ and

B.6 $$\begin{aligned} \partial _1 \Phi ^\sigma = \Big ( \frac{BR^2}{h} \Big )^{ \frac{\Theta _0}{4}}\exp \Big ( - \frac{\varphi _\sigma }{h} \Big ) \Big ( \frac{x_1}{|x|} \partial _r u_h - h^{-1} \partial _1 \varphi _\sigma u_h \Big ). \end{aligned}$$

Using these formulas we find

$$\begin{aligned} w_\ell ^{\sigma \sigma '} = h^2 \Big ( \frac{BR^2}{h} \Big )^{ \frac{\Theta _0}{2}} \int _{{\mathbb {R}}} e^{- \frac{s_h^{\sigma \sigma '}( 0, x_2)}{h}} u_h(M(x_2)) \omega (x_2) \textrm{d}x_2, \end{aligned}$$

with $$M(x_2) = \sqrt{x_2^2 + \ell ^2/4}$$ and

$$\begin{aligned} \omega (x_2)&= \frac{\ell }{\sqrt{\ell ^2 + 4 x_2^2}} \partial _r u_h(M(x_2)) + \Big ( \frac{m_{\sigma '}}{\ell /2 + i x_2} - \frac{B \ell }{4h} \Big ) u_h(M(x_2)) \\ s_h^{\sigma \sigma '}(0,x_2)&= \frac{B \ell ^2}{8} - \frac{BR^2}{2} + \frac{B x_2^2}{2} - h(m_\sigma + m_{\sigma '}) \log \Big ( \frac{\ell /2 + i x_2}{R}\Big ) + \frac{iB \ell x_2}{2}. \end{aligned}$$

The main properties of the function $s_h^{\sigma \sigma '}$ and its principal part $s_0$ are summarized in Lemma 17. In particular it has a unique critical point in the lower half complex plane at $x_2^* = -i \sqrt{\frac{\ell ^2}{4} - R^2}$. Since the involved functions are analytic, we can make a complex translation $x_2 \mapsto x_2 + x_2^*$ to obtain

$$\begin{aligned} w_\ell ^{\sigma \sigma '} = h^2 \Big ( \frac{BR^2}{h} \Big )^{ \frac{\Theta _0}{2}} \int _{{\mathbb {R}}} e^{- \frac{s_h^{\sigma \sigma '}( 0, x_2 + x_2^*)}{h}} u(M(x_2 + x_2^*)) \omega (x_2 + x_2^*) \textrm{d}x_2. \end{aligned}$$

We estimate this integral using the Laplace method. We give a few details due to the complexity of the phase involved. Since the phase is minimal at $x_2=0$, and using (2.7), we can restrict the integral to $|x_2| \le C h^{1/4}$. We then rescale with $x_2 = \sqrt{\frac{hN}{B(1-N)}} t$, where we define $N = \frac{4R^2}{\ell ^2}$. With this notation we have

$$\begin{aligned} M(x_2 + x_2^*) = R - i t \sqrt{\frac{h}{B}} + {\mathscr {O}}(h), \end{aligned}$$

and therefore

$$\begin{aligned} w_\ell ^{\sigma \sigma '}= &   - \frac{ h^{2}}{2} \Big ( \frac{BR^2}{h} \Big )^{ \frac{\Theta _0+1}{2}} e^{-s_h^{\sigma \sigma '}(0,x_2^*) /h } \\  &   \quad \int _{\lbrace | t| <C h^{- 1/4} \rbrace } e^{- \frac{1-\sqrt{1-N}}{\sqrt{1-N}}(t^2 +2it \sqrt{\Theta _0})} u_h \Big (R - i t \sqrt{\frac{h}{B}} \Big )^2 \big ( 1 + o(1) \big )\textrm{d}t. \end{aligned}$$

Here we used Lemma 17 to estimate the phase $s_h^{\sigma \sigma '}$, and Lemma 7 to show that, in $\omega$, the term in $\partial _r u_h$ is smaller than $h^{-1} u_h$. Now note that in this regime $|t|< C h^{-1/4}$, the estimate (2.8) remains with $-it$ instead of *t*. Therefore,

$$\begin{aligned} w_\ell ^{\sigma \sigma '} = - \frac{K_0^2 B h}{2} e^{-s_h^{\sigma \sigma '}(0,x_2^*) /h } \int _{\lbrace | t| <C h^{- 1/4} \rbrace } e^{- \frac{1-\sqrt{1-N}}{\sqrt{1-N}}(t^2 +2it \sqrt{\Theta _0})} v(-it) ^2 \big ( 1 + o(1) \big )\textrm{d}t. \end{aligned}$$

Also note that $v(-it)$ belongs to the Schwarz class, since it is the Fourier transform of a Schwarz function. Thus, the integral is asymptotically equal to a constant, which only depends on *N*. We obtain a constant $\tilde{C}_\ell \ne 0$ such that

B.7 $$\begin{aligned} w_h^{\sigma \sigma '}(\ell ) = {\tilde{C}}_\ell h e^{-\frac{s_h^{\sigma \sigma '}(0,x_2^*)}{h}} ( 1+ o(1)). \end{aligned}$$

The critical value of $s_h$ is equal to

$$\begin{aligned} s_h^{\sigma \sigma '}(0,x_2^*) = S_h(\ell ) - h ( 2 {\mathcal {C}}_2 + m_\sigma + m_{\sigma '} - 2 \xi _h) \ln (K_\ell ), \end{aligned}$$

where we recall that $m_- = \xi _h - e_0 + o(1)$ and $m_+=m_-+1$. The result follows with a new $C_\ell$. $\square$

## Appendix C. Error Terms

We recall the order of the error terms encountered,

$$\begin{aligned} {\mathcal {E}}_{\varepsilon ,L} = e^{- \frac{2S_h(L)}{h}} + e^{-\frac{2d(L-R-4\eta )}{h}}+ e^{-\frac{S_h(L(1+\varepsilon ))}{h}} + e^{-\frac{d(L-R-4\eta ) + d(\varepsilon L + R + 2 \eta )}{h}}. \end{aligned}$$

We prove here that these errors are indeed small enough.

### Lemma 21

If $L > \max (6R, \frac{4}{\varepsilon ^2} R)$ and if $\eta$ is small enough, then there exists $\kappa >0$ such that

$$\begin{aligned} {\mathcal {E}}_{\varepsilon ,L} = {\mathscr {O}} \Big ( e^{-\frac{S_h(L)(1+ \kappa )}{h}} \Big ). \end{aligned}$$

### Proof

We recall that $S_h(\ell ) = S_0(\ell ) - \sqrt{h} S_1(\ell ) + {\mathscr {O}}(h)$ with

$$\begin{aligned}  &   S_0(\ell ) = \frac{B \ell }{4}\sqrt{\ell ^2-4R^2} - BR^2 \ln \Big ( \frac{\ell + \sqrt{\ell ^2-4R^2}}{2R} \Big ), \\  &   S_1(\ell ) = 2\sqrt{\Theta _0 BR^2} \ln \Big ( \frac{\ell + \sqrt{\ell ^2 - 4R^2}}{2R}\Big ). \end{aligned}$$

Moreover, the function $S_0$ can be rewritten as

C.1 $$\begin{aligned} S_0(r) = 2 BR^2 \int _1^{r/2R} \sqrt{u^2-1} \textrm{d}u, \end{aligned}$$

and the Agmon distance as

C.2 $$\begin{aligned} d(r) = \frac{BR^2}{2} \int _1^{r/R} \Big ( t - \frac{1}{t} \Big ) \textrm{d}t. \end{aligned}$$

In particular, $S_0$ and *d* are positive increasing functions on $[R,\infty )$. Therefore, it is enough to prove that

C.3 $$\begin{aligned} 2 d(L-R) > S_0(L), \end{aligned}$$

and

C.4 $$\begin{aligned} d(L-R) + d(\varepsilon L + R) > S_0(L). \end{aligned}$$

To prove these, we use $\sqrt{u^2-1} < u$ in (C.1) and $t - \frac{1}{t} > t-1$ in (C.2). This provides us the upper bound

C.5 $$\begin{aligned} S_0(L) \le \frac{BL^2}{4} - BR^2, \end{aligned}$$

and the lower bound

C.6 $$\begin{aligned} d(r) \ge \frac{B}{4} (r-R)^2. \end{aligned}$$

Thus (C.3) is satisfied when

$$\begin{aligned} \frac{B}{2} (L-2R)^2> \frac{BL^2}{4} - BR^2, \quad \mathrm{{i.e.}} \quad \frac{L^2}{4} - 2LR+ 3R^2 >0, \end{aligned}$$

for which it is enough that $L > 6R$. The other inequality (C.4) is satisfied when

$$\begin{aligned} \frac{B}{4} (L-2R)^2 + \frac{\varepsilon ^2 BL^2}{4} > \frac{BL^2}{4} - BR^2, \quad \mathrm{{i.e.}} \quad LR < \frac{\varepsilon ^2 L^2}{4} + 2R^2, \end{aligned}$$

for which it is enough that $L > \frac{4}{\varepsilon ^2} R$. $\square$

## Footnotes

1 When $V=\lbrace \alpha \rbrace$, we write ${\mathscr {L}}_{h}^\alpha$ instead of ${\mathscr {L}}_{h}^{\lbrace \alpha \rbrace }$ for simplicity.

2 Here and in the rest of the article $\alpha \wedge x = \alpha _1 x_2 - \alpha _2 x_1$ is the standard wedge product on ${\mathbb {R}}^2$.

3 With slight abuse of notation, we still denote by ${\mathscr {L}}_{h}$ the operator in radial coordinates.
